# Expanding the scope of practice for nurses: possibilities in five Latin American countries^1^

**DOI:** 10.1590/1518-8345.8037.4819

**Published:** 2026-06-15

**Authors:** Bruna Moreno-Dias, Livia Angeli-Silva, Clara Aleida Prada-Sanabria, Tiago Parada Costa Silva, Silvia Helena de Bortoli Cassiani, Eduardo Benjamin Puertas-Donoso

**Affiliations:** 1 Organización Panamericana de la Salud, Departamento de Sistemas y Servicios de Salud, Washington, DC, United States of America.; 2 Universidade Federal da Bahia, Escola de Enfermagem, Salvador, BA, Brazil.; 3 Universidade Estadual de Feira de Santana, Departamento de Saúde, Feira de Santana, BA, Brazil.

**Keywords:** Advanced Practice Nursing, Primary Care Nursing, Nurse’s Role, Graduate Nursing Education, Health Workforce, Health Systems.

## Abstract

**Objective:**

to analyze, from a comparative perspective, the training and regulation of nurses’ work and the proposals for the implementation of the advanced practice nurse role in selected Latin American countries.

**Method:**

a descriptive comparative case study was conducted to understand the training and regulation of nursing work in five countries: Brazil, Chile, Colombia, Costa Rica, and Mexico. Data were collected in five focus groups with key informants from each country. The content was systematized in an analytical matrix that was validated by the participants.

**Results:**

the countries are at different stages of debate and implementation of expanding the scope of practice. The primary care level is considered a potential space for the performance of this role. In addition, master’s programs constitute a training opportunity for these professionals. The exchange of international experiences and working groups have been initiatives to discuss the role and consider the specific characteristics of each country in its implementation process.

**Conclusion:**

there are interest and initiatives to expand the role of nurses in Latin America; however, there is still a long way to go in terms of training, scope of practice, regulation, and the labor market.

## Highlights

**(1)** There is an underutilization of the contributions and skills of nurses. **(2)** The regulatory framework should be updated from an interprofessional perspective. **(3)** Working groups and exchanges of experiences have been initiatives to discuss advanced practice nursing (APN). **(4)** The development of training programs in some countries has fostered its implementation. **(5)** A broad debate with different stakeholders and sectors is essential to advance APN.

## Introduction

Nurses constitute an important contingent in the health systems of the countries of the Region of the Americas and are recognized as essential professionals for universal access to and coverage of health care^1^.

Inequalities in the distribution of professionals, associated with the need to expand the population’s access to health services and quality professionals, have contributed, in some countries, to the development of professionals with expanded roles, such as the advanced practice nurse (APN), with the aim of improving access to health services, guaranteeing universal coverage, promoting the sustainability of health systems, and meeting global health needs^1^ ^-^ ^3^.

The Pan American Health Organization (PAHO) considers APNs as professionals with postgraduate training who, integrated into interprofessional teams, contribute to the management of care for patients or users with acute, mild illnesses and diagnosed chronic disorders, according to clinical protocols or guidelines. The expanded professional practice is characterized by a degree of autonomy in decision-making, which includes the diagnosis and treatment of patients’ disorders^3^.

In some countries, especially those with stronger economies and health systems with greater population coverage, the expansion of nurses’ roles has been positively evaluated, especially in the continuity of care, cost reduction, and user satisfaction^4^ ^-^ ^6^.

In the Americas, the experiences of Canada and the United States are well established. Latin America, in turn, is in an incipient and diversified phase with regard to the understanding, training, and regulation of APNs, despite initiatives aimed at their development^2^ ^-^ ^3^.

The debate on the regulatory framework, the development of competencies, and the working conditions of professionals with an expanded scope of practice represents an opportunity for the countries of the Americas Region, as highlighted in PAHO’s “Policy on the Health Workforce 2030: Strengthening Human Resources for Health to Achieve Resilient Health Systems”^7^.

The expansion of the scope of practice depends on the configuration of nursing in each country, and therefore, knowledge of the different realities is fundamental for the advancement of policies. Understanding the similarities and differences in nursing and its role in the different health systems of Latin American countries will help to indicate the scope of practice that best responds to the health needs of each country.

Therefore, this article aims to analyze, from a comparative perspective, the training and regulation of nurses’ work and the proposals for the implementation of the role of advanced practice nurses in selected Latin American countries.

## Method

### Study design 

A descriptive comparative case study^8^ to understand the formation and regulation of nursing work.

### Setting

Five Latin American countries were selected: Brazil, Chile, Colombia, Costa Rica, and Mexico, as they are considered favorable settings for the implementation of advanced practice nursing.

### Participants

Key informants representing three segments were considered eligible for the study: nursing associations and councils; universities or research institutions specializing in the topic; and ministries of health responsible for policies related to health personnel. The composition of each group took into account the presence of at least one representative from each sector. Participants were identified with the support of PAHO offices in the countries included in the study, as well as through collaboration with professional associations and ministries of health, by sending official memoranda. After this identification, potential participants received detailed information about the research and a formal invitation to participate via email. Sixty-five potential participants were identified, of whom 39 participated (4 from Brazil, 14 from Chile, 6 from Colombia, 7 from Costa Rica, and 8 from Mexico). Individuals who, despite being nominated, did not respond to the invitation were excluded.

### Data collection

The data were collected through five focus groups, one in each country, conducted between June and July 2023. The focus groups were moderated by two researchers specializing in the topic and with experience in this type of study, who used a script for moderating the discussion group and the following trigger questions:

How is the training of graduate nurses in your country (at the undergraduate and postgraduate levels)?

What is the role played by graduate nurses in the health system of your country?

What activities are carried out by graduate nurses in the first level of care/Primary Health Care?

Are there any initiatives or discussions about expanding the role of advanced practice nurses in the first level of care/Primary Health Care in your country?

If there are initiatives or proposals to expand the role of nurses, what training has been required? What are the forms of regulation and how would the job placement occur?

Each focus group lasted approximately two hours and was conducted through a virtual platform. All sessions were recorded and transcribed in full. In addition, searches were conducted for relevant documents on official websites of the health authorities of the countries included in the study, in indexed databases, and in the documents cited by the participants of the focus groups.

### Data analysis

The content of the focus group transcripts was subjected to thematic analysis. The results were systematized into a thematic matrix with four categories: the role of nurses; undergraduate and postgraduate training; the regulation of the scope of practice; and the expansion of the scope of practice. Once the research team completed the matrix, it was validated by the study participants in a virtual meeting held in September 2023, lasting one hour and with the participation of 14 people who had previously participated in the focus groups from each country: 2 from Brazil, 4 from Chile, 2 from Colombia, 3 from Costa Rica, and 3 from Mexico.

### Ethical considerations

The research protocol was reviewed and approved by the Pan American Health Organization Ethics Review Committee (PAHOERC) with opinion PAHOERC.0622.01. Consent to participate in the study was recorded through an electronic form shared with participants at the beginning of the focus group.

## Results

The study sample consisted of 39 participants, mostly women (62%). A relatively balanced distribution was observed regarding institutional origin: 38% came from professional associations, councils, or federations, 33% from ministries of health or social security, and 28% from academic institutions. This composition made it possible to gather diverse technical, scientific, political, and strategic perspectives, as well as complementary views on the training processes, scope of practice, policies, and governance of the nursing workforce.

In Brazil, the four participants were women: two representatives from professional associations and councils, one from the Ministry of Health, and one from an academic institution.

In Chile, of the 14 participants, seven were women; six came from academic institutions, five from professional councils or federations, and three from the Ministry of Health.

In Colombia, the six participants were women: three from professional associations, two from the Ministry of Health, and one from an academic institution.

In Costa Rica, of the seven participants, five were men; four came from public services (one from the Ministry of Health and three from the Costa Rican Social Security Fund), one from the professional college, and one from an academic institution.

In Mexico, of the eight participants, five were women; three came from the Ministry of Health, three from professional councils or federations, and two from academic institutions.

The results obtained in the focus groups are presented below by thematic categories. It is important to note that, whenever the information comes from document analysis, the corresponding reference is included.

### Nurses’ role

The five countries share the predominance of nurses in healthcare services, and they also play a significant administrative role; furthermore, they occupy different positions in the organizational structure of the system. However, their presence in decision-making spaces and strategic positions remains a challenge.

In addition, there are challenges for nurses to be recognized for their work. The subcontracting of nurses for roles below their level of training was a factor pointed out in Colombia, Costa Rica, and Mexico. This prevents a significant number of professionals from fully exercising the functions for which they are trained and qualified, which could contribute to achieving a more effective health system.

Within the scope of Primary Health Care (PHC), common activities carried out by nurses include immunization, coordination of programs aimed at controlling communicable and non-communicable diseases, as well as administrative tasks. A marked difference is observed in health actions aimed at women, which in Chile are the responsibility of midwives and, in Costa Rica, the exclusive responsibility of midwifery nurses. In general terms, the role of nurses is similar; however, the scope of their functions and their ability to solve health problems are related to the level of organization of PHC in each country.

In all five countries, the need for nurses in PHC to work more closely with the community, focusing on disease prevention and the social determinants of health, is highlighted.

The focus group in Mexico indicated that nurses receive training to work in PHC, but the labor market does not favor this role. In Colombia, the health system does not encourage this training, and there are few practice settings at the primary care level, despite the potential of these professionals to work in the community. On the other hand, Costa Rica points to the weakness of training for primary care, while Chile indicates the need to discuss the role of nurses at this level of care. In addition to these elements, factors that limit the work of nurses were identified, such as the excessive amount of administrative tasks, which hinder care-related actions, and the inadequacy of undergraduate training in the scope of practice required by primary health care.

Despite these limitations in PHC in these countries, some potential strengths were noted. In the case of Brazil, the leadership role that nurses play in the interprofessional team stands out, possibly the area in which these professionals have the greatest prominence and autonomy and, in some regions of the country, they can rely on protocols that allow them to expand the scope of their clinical practice.

In Chile, PHC is a space where the work of nurses has historically been recognized, as they have held leadership positions in health institutions and been responsible for relevant programs, such as immunization programs. At the same time, it is the level of care that most favors interprofessional collaboration.

In Colombia, a care model with a greater emphasis on prevention is proposed, and from this, the proposal arose for nurses to work more closely with the community. It is recognized that the territory and communities offer leadership opportunities for nursing to address new health problems and transform care.

In Costa Rica, nurses play an essential role in the first contact of users with the health system. Although it is noted that some care actions are delegated to nursing assistants, the managerial function of nurses is highlighted, as well as the need to expand their leadership in the community. This community work is also emphasized in Mexico, where good results are observed when it is carried out by nursing professionals and is considered a transformative element of the health reality and community development.

### Education at the undergraduate and postgraduate levels

The training of nurses in the five countries is at the university level; although there are similar aspects, certain particularities are also observed, as indicated in Figure 1.

**Figure 1:** Characteristics of undergraduate nursing education in Brazil, Chile, Colombia, Costa Rica, and Mexico, 2023

**Country**	**Characteristics**
**Brazil**	National curriculum guidelines adapted to regional needs and epidemiological profiles^11^. Duration of 4 to 6 years, with a minimum of 3,600 hours^12^. Theoretical and practical activities are developed throughout the entire training^11^. Internship in real working conditions in the last year (with an equitable distribution of workload between hospital and Primary Health Care areas)^11^. Of the courses, 11.2% are offered in public institutions and 88.8% in private institutions^13^.
**Chile**	Duration of 5 years (10 semesters)^14^. The content of the training is defined by the universities with the objective of fulfilling the four functions of nurses: care, education, research, and community work. Undergraduate curricula include fundamental subjects such as: 1) Humanities (anthropology, philosophy, ethics, psychology, among others); 2) Basic sciences (morphology, physiology, pathophysiology, pharmacology, biochemistry, among others); 3) Disciplinary sciences (nursing models and theories, nursing care process, nursing care in different stages of the life cycle, management and administration in nursing, among others)^15^. There are no exact numbers of public and private institutions.
**Colombia**	Duration of 8 to 10 semesters^16^. The training is organized into three components: 1) Social sciences and natural sciences; 2) Specific disciplinary area of nursing practice (70 to 80%); 3) Interface with other areas of knowledge. One year of social work at the end of the course (with a choice of different areas). 37% are in public institutions and 63% in private institutions^16^.
**Costa Rica**	Duration of 4 to 5 years. There is no unified curriculum: training varies considerably between universities, with greater or lesser emphasis on care, education, or primary care. The country has regulatory bodies for higher education. The training is structured in a theoretical phase followed by a supervised practical phase. The country has one public university and seven private universities.
**Mexico**	Two types of courses are offered: 1) Bridging course: aimed at those with technical training; lasting between one and two years, depending on the program; 2) Four-year undergraduate degree. At the end of the undergraduate degree, an additional year of social work in primary health care is required. The training is governed by the Higher Education Law (undergraduate and postgraduate). The exact number of public and private institutions is not available.

In Brazil, there is concern about the exponential growth in the number of private universities and the adoption of distance education, which does not always provide the quality of training required by professionals and the population. Furthermore, the weakness of the evaluation process for the creation and continuous operation of courses is also a concern^9^.

In Chile, the nursing profession has been regulated by law since 1997^10^. The Nursing course is at the university level, but it is not recognized as such in the education law. A set of regulations on the accreditation and licensing of Nursing courses is currently under consideration by the National Accreditation Commission. In addition, promoting training less centered on the biomedical paradigm remains a challenge for many universities.

Colombia has solid clinical training, due, among other factors, to the practice settings provided by the system, where job opportunities are predominantly in hospitals. The health system, focused on specialized and subspecialized care, ultimately shapes the curricula, training, and framework of competencies and skills.

In Costa Rica, the variety of study plans continues to ensure that the biomedical paradigm predominates in many institutions. This difficulty in training has impacted the work of nurses in the Costa Rican health system.

As in other countries, the biomedical training model constitutes a limiting aspect in Mexico for the training of nurses, which impacts the interprofessional approach.

Postgraduate education is more diverse than undergraduate education, both in the development of programs and in the role played by specializations in integrating nurses into the labor market. Figure 2 presents an overview of postgraduate education in these countries.

**Figure 2:** Types and characteristics of postgraduate education in Brazil, Chile, Colombia, Costa Rica, and Mexico, 2023

Program	Brazil	Chile	Colombia	Costa Rica	Mexico
**Specialization**	Minimum of 360 hours; courses in various areas	1 year; clinical focus postgraduate degrees or specialization courses	46 areas 1 year/1 and style="padding: 10px; border:1px solid black" a half years	There are none	Courses in various areas of nursing care
**Specialization in residency format (theoretical-practical modality)**	2 years; minimum of 5,760 hours	There are none	There are none	There are none	There are none
**Academic Master’s degree**	54	Unidentified number	Unidentified number	Unidentified number	Unidentified number
**Professional or advanced master’s degree**	24	Total number of professional master’s degrees not identified; two master’s degrees for APN*.	Unidentified figure	Unidentified figure	Total number of professional master’s degrees not identified; one master’s degree for APN*.
**Academic PhD**	41	2	3	There are none	Unidentified number
**Profissional Doctorate degree**	2	There are none	There are none	There are none	There are none

*APN = Advanced practice nurse

Brazil has the largest number of master’s and doctoral programs. The courses are standardized and evaluated by the Coordination for the Improvement of Higher Education Personnel (CAPES) and follow the same rules for all areas of knowledge. Most postgraduate courses are offered at public universities and are free of charge. Through CAPES and other funding agencies, it is possible to obtain scholarships for this training. The country has specialization programs in the form of residencies, specific to nursing (uniprofessional) or multiprofessional, with scholarships. In addition, there are specializations in various areas of nursing and related fields. This is the most accessible type of postgraduate education, but also the least regulated. As a result, and due to the expansion of private education in the country, this modality has intensified, with the creation of a large number of courses, especially in the distance learning modality.

Chile has master’s degrees in nursing oriented towards research and master’s degrees in health sciences or of an interprofessional nature to which nurses can have access. More recently, a master’s degree focused on clinical practice was created, which confers an academic title and is aimed at training advanced practice nurses (APNs) in the area of ​​oncology. Regarding doctoral degrees, there are two programs in nursing. The recognition of specialties depends on the regulation of the Ministry of Health for the health area, which does not include nursing specialties. In this case, the scope of practice is not expanded, and nurses continue to perform the same functions, only with greater knowledge and, consequently, without salary differences.

Colombia has master’s and doctoral programs; this training is considered solid, but there is the possibility of expanding it, including practice in public health settings. Regarding specializations, it was pointed out that nurses face difficulties in pursuing postgraduate studies due to the cost and obstacles in balancing work and family life; furthermore, there is a lack of interest due to the lack of recognition of the specialization.

Costa Rica has master’s programs in nursing at public and private institutions. The College of Nurses of Costa Rica has regulations for the registration and recognition of postgraduate courses, covering both courses offered in the country and those taken abroad. There is no doctoral program in nursing in the country, but there are courses in related areas such as education, culture, and other areas pursued by nurses. Another challenge is the governmental recognition of postgraduate courses, which only recognizes those in mental health and psychiatry, and gynecology and obstetrics; however, the title does not always translate into higher salaries, and the training involves a cost.

In Mexico, master’s and doctoral programs are offered at public and private universities; in addition, some specializations have been converted into master’s degrees, but courses that confer the title of specialist through professional certification are still offered. Postgraduate education does not always lead to better salaries, but the specialization course is a requirement for access to some jobs.

### Regulation of the scope of practice

In all countries, legislation is applied at the national level; in Mexico, there is general national legislation, although the scope of professional practice may vary among the health subsystems and their institutions, as well as among the states of the federation. The professional registration procedure and the regulatory instruments that govern nursing practice are presented in Figure 3.

**Figure 3:** Professional registration and regulatory instruments governing the practice of nursing in Brazil, Chile, Colombia, Costa Rica, and Mexico, 2023

**Country**	**Registro profissional e instrumentos normativos**
**Brasil**	Professional registration: Federal Council of Nursing (Brazil) Regulatory instruments: Law on professional practice, No. 7.489/1986^17^, Code of professional ethics^18^; in addition to resolutions, decisions and normative opinions of the Federal Council and the System of Regional Nursing Councils^19^, as well as ordinances, decrees and other normative documents of the Ministry of Health and health secretariats.
**Chile**	Professional registration: College of Nurses of Chile^20^ Regulatory instruments: Law 19,536/1997 - recognizes the role of the nurse in the Chilean Sanitary Code; Law 19,937/2004 - incorporates “care management” into the structure of State-dependent Health Services, understanding it as the role of the nurse^21^ ^-^ ^22^; and the Code of Ethics - 2008^20^.
**Colombia**	Professional registration: National Single Nursing Registry at the National Association of Nurses of Colombia and, at the national level, the Collegiate Organization of Nursing issues the National Identification Card based on registration in the National Single Registry of Human Resources in Health. Regulatory instruments: Professional regulation law - Law 266/1996^23^; Law 911/2004 - National Court of Ethics in Nursing^24^.
**Costa Rica**	Professional registration: College of Nurses of Costa Rica. Regulatory instruments: Organic Law of the College of Nurses (Law 2343/1959) and Law 7085/87 - establishes the different levels of nurses that exist in health institutions, depending on the complexity of the nursing departments or services and the volume of work.
**Mexico**	The registration and licensing of professionals are carried out with the Mexican government. In 2000, nursing certification was established (a voluntary procedure that does not replace the license, and the lack of certification does not imply the loss of the license). There are two organizations officially authorized for certification: the Mexican Council for Nursing Certification and the Mexican College of Licensed Nurses^25^. Regulatory instruments: General Law of Professions (1945) and there are the Official Mexican Standards (NOM) that establish the areas of nursing practice in the national territory^25^.

Another element discussed was the performance of graduate nurses as nursing assistants or technicians, a practice present in Costa Rica and Mexico, while in Brazil it is not possible for a nurse to act as a nursing assistant or technician unless they have completed the assistant or technician course and are registered at that level.

### Initiatives to expand the scope of practice for nurses in primary health care services.

The context of expanding the scope of nursing practice in the countries analyzed is heterogeneous and depends on the stage of development of nursing practice and education in each country, as well as on the strategies for regulating professional practice, the characteristics of the health systems, and the provisions and regulations of health institutions. The main aspects by country are presented below.

#### Brazil

In Brazil, the Federal Council of Nursing (COFEN) and the Brazilian Nursing Association created working groups to promote discussions on the topic and develop documents. In 2023, the Ministry of Health created a working group composed of different nursing associations and specialties, which assumed a leading role in the debates on nursing practice and the possibilities of expanding the role of nurses. Research on the topic has also been encouraged, and several articles have been published in different scientific journals.

Since 2021, COFEN has sought to exchange international experiences to support the processes of expanding the scope of practice. The organization established alliances with CAPES through financial support for existing professional master’s programs in Brazil and promoted the development of proposals for master’s degrees in APN.

The professional practice law guarantees nurses the possibility of expanding their scope of practice in public health programs, through appropriate protocols^17^. Considering that the country adopts the model of self-regulation of the profession by professional councils, this favors the updating of resolutions and normative acts that are considered necessary to support professional practice.

Over the years, an expansion of the nurse’s role has been observed in various areas, through several specialties, such as neonatology, midwifery in secondary care, and diagnostic support through ultrasound. As in primary health care, the role of these professionals has been expanded through the development of clinical protocols in various areas, which allows us to consider that advanced practices are already being developed. Despite this scenario, challenges remain for expanding the scope of practice: among them, the training of specialists, regulation, the labor market, and the acceptance of APNs by other professionals, health services and providers, and the general population.

Among the elements that should be analyzed are whether the APN constitutes another professional category and how it would relate to nurses, who already have an expanded scope of practice in Brazil, and the suitability of the training model, given that there are divergent opinions on training at the master’s or residency level.

#### Chile

In Chile, the implementation of APN was carried out through discussions with international stakeholders and initiatives that used the participatory process for advanced practice nursing, patient-centered and evidence-based (PEPPA)^26^, which develops the program based on the country’s needs and identifies the interlocutors for its insertion in clinical settings.

The Universidad Católica de Chile took on the challenge of creating an APN training program and, from the beginning, considered it necessary to address the political aspects to critically train nurses. However, attempts to expand the role in primary health care met with resistance from family and community physicians. At the same time, conditions were created to expand the role of nurses in oncology based on the National Cancer Plan 2018-2028, which includes the creation of a postgraduate program for non-medical professionals, aimed at expanding the scope of practice^27^. Two master’s programs in APN in this area were then created.

APN in oncology was facilitated in the clinical environment, since nurses were already integrated and performing established functions, so they continued in the same positions and functions, but with solid training, which resulted in improvements in patient care. However, remuneration constitutes the main challenge, as no salary increases were made for this new role, nor were specific positions or functions defined.

There are initiatives by the Ministry of Health for the recognition of specialties, including APN. However, conflicts persist in the country regarding the regulation of professional practice, requiring a broader debate on training and practice in health.

Despite these challenges, the prospects for expanding the scope of practice remain promising, and there has been extensive debate on this matter. It is proposed as a premise that it is fundamental to consider the PHC approach.

#### Colombia

There are different initiatives to develop the role of APN at different levels of care, which have the support of the Ministry of Health. Seven meetings have been held on advanced practice nursing, with the participation of people who have supported experiences in other countries.

Similarly, the health system reform is oriented towards strengthening preventive actions and has sought to establish a regulatory framework that includes funding for advanced practice training. The priority is to begin training in public universities and define the professional profile of the APN in PHC.

The position of the broad advanced training group, composed of directors of master’s and doctoral programs and members of the board of the Colombian Association of Faculties and Schools of Nursing, maintains that the level of training for APN should be a master’s degree with a focus on advanced practice.

Despite the initiatives, the challenges of implementation lie in several aspects, including the availability of and access to courses, accompanied by a Ministry regulation that allows for the development of the practice and supports postgraduate studies.

Another difficulty is the low recognition of postgraduate training, which does not always imply recognition by peers and society, nor does it translate into higher salary levels. There are other aspects that favor training at the master’s level, such as the availability of scholarships that encourage training. An economic incentive has also been sought through the monthly educational subsidy provided for in the Residents Law, currently exclusive to physicians. Residencies, as part of postgraduate nursing education, would favor the improvement of clinical practice through an immersion process and with salaries that allow dedication to this training.

More immediately, progress has been made in defining the profile with the aim of guiding the implementation of APN. There have also been advances in the curricular and organizational processes of the courses.

#### Costa Rica

In 2017, the College of Nurses of Costa Rica held the first meeting to begin the process of analyzing and defining the line of work for the development of APN. In 2019, the first national APN workshop was held, through an intersectoral working group that involved the academic sector and sought to define short- and medium-term actions.

Universities have initiatives to analyze the possibility of offering advanced practice training with groups of leading nurses in Central America. Regarding nursing practice, the need to overcome the shortage of nurses and deficient infrastructure is highlighted, as well as the need to consolidate the leadership of nurses.

Costa Rica also has a high percentage of graduate nurses subcontracted in roles below their level of training. In this debate process, issues that require further in-depth analysis in the country were identified, such as the regulation of professional practice.

It is necessary to consider a professional profile with advanced functions that can, in fact, respond to the health needs of the population; which necessarily implies the training process and how advanced practice proposals will be outlined. Therefore, to achieve structural changes, it is necessary to have a professional nursing profile that is not only qualified from the point of view of care and system management, but also with political competencies that position nurses at decision-making tables and allow them to influence the construction of broader policies.

#### Mexico

In 2019, a working group was established to expand the scope of nursing practice, led by the Pan American Health Organization (PAHO), the National Autonomous University of Mexico, the Autonomous University of Xochimilco, and the Ministry of Health, with the participation of the College of Nurses, the Permanent Nursing Commission of the Ministry of Health, and universities.

Among the products is the development of a Mexican normative framework of nursing competencies, with six domains and 68 competencies for the expanded list of functions of nurses with experience in PHC. Mexico’s strategy to enhance the work of nurses has two aspects: 1) expanding the functions of those who have worked for two years or more in PHC, insofar as current regulations allow; 2) establishing the role of the APN in the country, which includes regulatory changes and master’s level training.

The Ministry of Health has assumed a central role in the entire process and is preparing to launch pilot experiences in several states of the Mexican Republic, in which these advanced practice nurses will be placed in health centers where they can perform expanded functions. To this end, proposals for APN training programs have been formulated based on international recommendations for master’s degrees and with a public health focus. The first master’s program was conducted between 2021 and 2022.

It should be noted that the debate surrounding the expansion of the role of nurses in Mexico also developed through parallel channels, as it involved other actors outside the health system.

Challenges remain to be overcome in this process, the main one being the undervaluation of graduate nurses. Specializations do not guarantee better salaries, which is why the requirement of a master’s degree for APNs is a cause for concern. It is also necessary to advance in the regulatory field, including considering the differences in the scope of already regulated practices among the different states.

## Discussion

The education and practice of nursing are heterogeneous in the contexts analyzed. The configuration of the scope of practice in the countries analyzed has been defined according to their specific realities. Despite similarities in the role of nurses, the scope of their actions, their recognition, and their influence remain disparate.

The concept of advanced practice and the motivations and interests for implementing it also vary between countries^28^. Similarly, an analysis of the situation of APN in Europe reveals significant variations in regulation, training, and scope of practice, with different levels of competence and autonomy in healthcare^29^ ^-^ ^30^.

The underutilization of the potential of nurses, their training, and their ability to meet the needs of the population and contribute to the management and formulation of health policies has been pointed out. This underutilization is manifested both in the scarcity of job opportunities and in the lack of recognition of these professionals to occupy strategic positions.

Because it is an incipient function in the countries under study, a weakness in terms of regulation is identified, making it necessary to establish a normative framework that formally recognizes the function, defines the criteria for licensing and registration renewal, as demonstrated by the experience of countries where the function is already consolidated^31^.

In this context, it is fundamental to establish the bases for defining the standards of practice and defending specific regulations^32^. Regulatory processes must be reviewed and updated based on the needs of local health systems and the creation of work spaces with social protection for workers.

Identifying and addressing regulatory barriers is essential for the full performance of APN practice and to expand its potential contributions to innovation and the transformation of care models^33^. However, the study findings indicate that deficient regulation prevails^34^, which may prevent this qualification from being immediately recognized and, furthermore, implies low market insertion, lack of role definition, salary differentiation, and disincentives to continue training, further aggravated by the fact that, in some countries, the economic investment required to access postgraduate programs is high.

It is important to ensure that nurses can fully perform their functions, in accordance with their regulatory framework, so that the work of these professionals has a significant impact. It is necessary to increase the number of job opportunities, especially in primary health care, guarantee decent working conditions, and review work processes to reposition the role of nurses within interprofessional health teams.

Investing in nursing and recognizing its role implies reducing gender inequalities, given that it is a predominantly female professional category, whose members face differences in working conditions, opportunities for advancement and leadership, and remuneration^35^.

Regarding capacity development, expanding the scope of practice requires coherent and quality training that provides professionals with broad knowledge and critical thinking about health conditions.

The results presented in this study indicate different possibilities for training programs, with an emphasis on master’s degrees or residency programs. International experience points to the adoption of formal training programs, with master’s degrees being the most frequent^31^.

It is recommended to expand training programs through public financial support that allows for greater dedication and reduces concerns about family income.

The countries analyzed in this study show differences in the availability of training programs. The supply and training capacity in specialization, master’s and doctoral programs should be optimized for the training of APNs; however, it is necessary to identify and define the competencies required for these professionals, which will facilitate the understanding, visibility, and implementation of APNs in health services^36^.

It is also important to discuss the role of this professional based on education and practice in interprofessional teams^37^ ^-^ ^38^. Expanding the role of nurses, combining tasks, and developing interprofessional teams are strategies that can be used to address the shortage of human resources for health and obtain positive results in improving healthcare for the population and in the satisfaction of professionals and users, especially in primary health care^37^. Evidence indicates that the integration of APNs contributes to improving care outcomes, expanding access, reducing healthcare costs, improving the quality of care, and increasing patient well-being^39^ ^-^ ^40^.

However, it is necessary to promote a shared understanding among healthcare teams, recognizing the role and contributions of APNs, their experience, and autonomy in healthcare^41^.

The effective and timely implementation of APNs should be based on health needs and not on workforce or professional imperatives. Although the implementation process may be prolonged, the experience of other countries where the role has been implemented reveals the importance of a broad debate and effective collaboration among policymakers, educators, employers, and professional associations to ensure that all initiatives align with the objectives during their implementation^30^ ^,^ ^42^.

Similarly, it is necessary to encourage the participation of these professionals in policy formulation and decision-making spaces. It is considered desirable to provide more information to society about their work, with an emphasis on the scientific evidence of their results. 

One limitation of this study lies in the nature of the data, which is based on the perceptions and experiences of selected participants. However, the inclusion of different key stakeholders in each country and triangulation with secondary data reinforce the robustness and credibility of the findings. Despite this limitation, the study makes relevant contributions by presenting a comparative analysis of various national contexts, which allows for the identification of common facilitating factors and challenges for expanding the scope of nursing practice in the Region of the Americas.

## Conclusion

The implementation of the advanced practice nurse role constitutes a real opportunity for countries to expand access to and coverage of healthcare, especially in primary healthcare. There are interest and initiatives to expand the role of nurses in Latin America; however, there is still a long way to go in terms of training, scope of practice, regulation, and the labor market.

The academic sector has played an important role in fostering the implementation of APN through the development of training programs. However, the experience of Latin American countries and other countries where APN is consolidated highlights the role of working groups and intersectoral actions and programs, with broad participation of various actors in the political dialogue for the effective and timely implementation of this role.

## Data Availability

Datasets related to this article will be available upon request to the corresponding author.
