# Supplementary material for: Expanding the scope of practice for nurses: possibilities in five Latin American countries
Source: Rev Lat Am Enfermagem. 2026 Jun 15;34:e4819. doi: 10.1590/1518-8345.8037.4819 (PMC13277355; doi:10.1590/1518-8345.8037.4819)
Supplement: Translation

# Translation: Ampliação do escopo de prática das enfermeiras: possibilidades em cinco países da América Latina^*^

**DOI:** 10.1590/1518-8345.8037.4820

**Authors:** Bruna Moreno-Dias, Livia Angeli-Silva, Clara Aleida Prada-Sanabria, Tiago Parada Costa Silva, Silvia Helena de Bortoli Cassiani, Eduardo Benjamin Puertas-Donoso

**Affiliations:** 1 Organización Panamericana de la Salud, Departamento de Sistemas y Servicios de Salud, Washington, DC, Estados Unidos da América; 2 Universidade Federal da Bahia, Escola de Enfermagem, Salvador, BA, Brasil.; 3 Universidade Estadual de Feira de Santana, Departamento de Saúde, Feira de Santana, BA, Brasil.

**Keywords:** Prática Avançada de Enfermagem, Enfermagem de Atenção Primária, Papel do Profissional de Enfermagem, Educação de Pós-Graduação em Enfermagem, Mão de Obra em Saúde, Sistemas de Saúde

## Abstract

**Objetivo:**

analisar, a partir de uma perspectiva comparada, a formação e a regulação do trabalho das enfermeiras e as propostas para a implementação da função das enfermeiras de prática avançada em países selecionados da América Latina.

**Método:**

estudo descritivo de casos comparados para compreender a formação e a regulação do trabalho de enfermagem em cinco países: Brasil, Chile, Colômbia, Costa Rica e México. Os dados foram coletados em cinco grupos focais com informantes-chave de cada país. O conteúdo foi sistematizado em uma matriz analítica que foi validada pelos participantes.

**Resultados:**

os países se encontram em diferentes fases de debate e implementação da ampliação do escopo de prática. O primeiro nível de atenção é considerado um espaço potencial para o desempenho dessa função. Além disso, os programas de mestrado constituem uma oportunidade de formação para essas profissionais. A troca de experiências internacionais e os grupos de trabalho têm sido iniciativas para debater a função e considerar as características específicas do país em seu processo de implementação.

**Conclusão:**

há interesse e iniciativas para ampliar a função das enfermeiras na América Latina; no entanto, ainda há caminho a percorrer em termos de formação, escopo de prática, regulação e mercado de trabalho.

## Destaques

(1) Há uma subutilização das contribuições e competências das enfermeiras. (2) O marco regulatório deve ser atualizado a partir de uma perspectiva interprofissional. (3) Grupos de trabalho e intercâmbio de experiências têm sido iniciativas para debater a EPA. (4) O desenvolvimento de programas de formação em alguns países tem fomentado a implantação. (5) Um amplo debate com diferentes atores e setores é essencial para fazer avançar a EPA.

## Introdução

As enfermeiras constituem um contingente importante nos sistemas de saúde dos países da Região das Américas e são reconhecidas como profissionais essenciais para o acesso e a cobertura universal da saúde^1^.

As desigualdades na distribuição dos profissionais, associadas à necessidade de ampliar o acesso da população a serviços de saúde e a profissionais de qualidade, contribuíram, em alguns países, para o desenvolvimento de profissionais com funções ampliadas, como a enfermeira de prática avançada (EPA), com o objetivo de melhorar o acesso aos serviços de saúde, garantir a cobertura universal, promover a sustentabilidade dos sistemas de saúde e atender às necessidades globais de saúde^1^ ^-^ ^3^.

A Organização Pan-Americana da Saúde (OPAS) considera as EPA como profissionais com formação de pós-graduação que, integradas em equipes interprofissionais, contribuem para a gestão do cuidado de pacientes ou usuários com doenças agudas, leves e transtornos crônicos diagnosticados, de acordo com protocolos ou diretrizes clínicas. O exercício profissional ampliado caracteriza-se por um grau de autonomia na tomada de decisões, que inclui o diagnóstico e o tratamento dos transtornos dos pacientes^3^.

Em alguns países, sobretudo naqueles com economias mais sólidas e sistemas de saúde com maior cobertura populacional, a ampliação das funções das enfermeiras tem sido positivamente avaliada, especialmente na continuidade do cuidado, na redução de custos e na satisfação dos usuários^4^ ^-^ ^6^.

Nas Américas, as experiências do Canadá e dos Estados Unidos estão consolidadas. Por sua vez, a América Latina encontra-se em uma fase incipiente e diversificada no que diz respeito à compreensão, à formação e à regulação das EPA, apesar das iniciativas direcionadas ao seu desenvolvimento^2^ ^-^ ^3^.

O debate sobre o marco regulatório, o desenvolvimento de competências e as condições de trabalho dos profissionais com escopo de prática ampliado representa uma oportunidade para os países da Região das Américas, como se destaca na “Política sobre a força de trabalho em saúde para 2030: fortalecendo os recursos humanos em saúde para alcançar sistemas de saúde resilientes” da OPAS^7^.

A ampliação do escopo da prática depende da configuração da Enfermagem nos países e, portanto, o conhecimento das diferentes realidades é fundamental para o avanço das políticas. A compreensão das semelhanças e diferenças da Enfermagem e de seu papel nos diferentes sistemas de saúde dos países da América Latina contribuirá para indicar o alcance da prática que melhor responda às necessidades de saúde de cada país.

Portanto, este artigo tem como objetivo analisar, a partir de uma perspectiva comparada, a formação e a regulação do trabalho das enfermeiras e as propostas para a implementação do papel das EPA em países selecionados da América Latina.

## Método

### Tipo de estudo 

Estudo descritivo de caso comparado^8^ para compreender a formação e a regulação do trabalho de Enfermagem.

### Cenário

Foram selecionados cinco países da América Latina: Brasil, Chile, Colômbia, Costa Rica e México, por serem considerados cenários favoráveis para a implementação da enfermagem de prática avançada.

### Participantes

Foram considerados elegíveis para o estudo informantes-chave representantes de três segmentos: associações e conselhos de enfermagem; universidades ou âmbitos de pesquisa especializados no tema; e ministérios da saúde, responsáveis pelas políticas relativas ao pessoal de saúde. A composição de cada grupo levou em conta a presença de ao menos um representante de cada setor. Os participantes foram identificados com o apoio dos escritórios da OPAS nos países incluídos no estudo, assim como por intermédio da articulação com associações profissionais e ministérios da saúde, mediante o envio de memorandos oficiais. Após essa identificação, os possíveis participantes receberam, por correio eletrônico, informações detalhadas sobre a pesquisa e um convite formal para participar. Foram identificados 65 potenciais participantes, dos quais participaram 39 (4 do Brasil, 14 do Chile, 6 da Colômbia, 7 da Costa Rica e 8 do México). Foram excluídas as pessoas que, apesar de terem sido nomeadas, não responderam ao convite.

### Coleta de dados

Os dados foram coletados por meio de cinco grupos focais, um em cada país, realizados entre junho e julho de 2023. Os grupos focais foram moderados por duas pesquisadoras especialistas na temática e com experiência nesse tipo de estudo, que utilizaram um roteiro para a moderação do grupo de discussão e as seguintes perguntas disparadoras:

Como é a formação das enfermeiras graduadas em seu país (em nível de graduação e de pós-graduação)?

Qual é a função desempenhada pelas enfermeiras graduadas no sistema de saúde de seu país?

Quais são as atividades desenvolvidas pelas enfermeiras graduadas nos serviços do primeiro nível de atenção/Atenção Primária à Saúde?

Existem iniciativas ou debates sobre a ampliação do papel das enfermeiras de prática avançada no primeiro nível de atenção/Atenção Primária à Saúde em seu país?

Se existem iniciativas ou propostas para ampliar o papel das enfermeiras, que formação tem sido exigida? Quais são as formas de regulação e como se daria a inserção laboral?

Cada grupo focal teve duração de aproximadamente duas horas e foi realizado por meio de uma plataforma virtual. Todas as sessões foram gravadas e transcritas integralmente. Além disso, foram realizadas buscas de documentos relevantes em sites oficiais das autoridades sanitárias dos países incluídos no estudo, em bases de dados indexadas e nos documentos citados pelos participantes dos grupos focais.

### Análise de dados

O conteúdo da transcrição dos grupos focais foi submetido a uma análise temática. Os resultados foram sistematizados em uma matriz temática com quatro categorias: o papel das enfermeiras; formação em nível de graduação e pós-graduação; a regulação do escopo de prática; e a ampliação do escopo de prática. Uma vez que a equipe de pesquisa concluiu a matriz, esta foi validada pelos participantes do estudo em uma reunião virtual realizada em setembro de 2023, com duração de uma hora e a participação de 14 pessoas que haviam participado previamente dos grupos focais de cada país: 2 do Brasil, 4 do Chile, 2 da Colômbia, 3 da Costa Rica e 3 do México.

### Considerações éticas

O protocolo de pesquisa foi analisado e aprovado pelo Comitê de Revisão Ética da Organização Pan-Americana da Saúde (PAHOERC) com o parecer PAHOERC.0622.01. O consentimento para participar do estudo foi registrado por meio de um formulário eletrônico compartilhado com os participantes no início do grupo focal.

## Resultados

A amostra do estudo foi composta por 39 participantes, em sua maioria mulheres (62%). Observou-se uma distribuição relativamente equilibrada quanto à origem institucional: 38% provinham de associações, conselhos ou federações profissionais, 33% de ministérios da saúde ou de seguridade social e 28% de instituições acadêmicas. Essa composição possibilitou reunir diversas perspectivas técnicas, científicas, políticas e estratégicas, bem como visões complementares sobre os processos de formação, o escopo de prática, as políticas e a governança da força de trabalho de Enfermagem.

No Brasil, as quatro participantes eram mulheres: duas representantes de associações e conselhos profissionais, uma do Ministério da Saúde e uma proveniente de uma instituição acadêmica.

No Chile, dos 14 participantes, sete eram mulheres; seis procediam de instituições acadêmicas, cinco de conselhos profissionais ou federações e três do Ministério da Saúde.

Na Colômbia, as seis participantes eram mulheres: três de associações profissionais, duas do Ministério da Saúde e uma proveniente de uma instituição acadêmica.

Na Costa Rica, dos sete participantes, cinco eram homens; quatro provinham dos serviços públicos (um do Ministério da Saúde e três da Caja Costarricense de Seguro Social), do colégio profissional e um de uma instituição acadêmica.

No México, dos oito participantes, cinco eram mulheres; três provinham do Ministério da Saúde, três de conselhos ou federações profissionais e dois de instituições acadêmicas.

A seguir, apresentam-se os resultados obtidos nos grupos focais por categorias temáticas. É importante destacar que, sempre que se tratar de informação proveniente da análise documental, inclui-se a referência correspondente.

### O papel das enfermeiras

Os cinco países compartilham o predomínio das enfermeiras nos serviços assistenciais, e também desempenham um papel administrativo relevante; além disso, ocupam diferentes funções na estrutura organizacional do sistema. No entanto, a presença em espaços de decisão e em posições estratégicas continua sendo um desafio.

Além disso, existem desafios para que as enfermeiras sejam reconhecidas pelo seu trabalho. A subcontratação de enfermeiras para funções aquém de seu nível de formação foi um fator apontado na Colômbia, na Costa Rica e no México. Isso impede que um número importante de profissionais exerça plenamente as funções para as quais estão formadas e qualificadas, o que poderia contribuir para alcançar um sistema de saúde mais eficaz.

No âmbito da Atenção Primária à Saúde (APS), entre as atividades comuns desenvolvidas pelas enfermeiras encontram-se a imunização, a coordenação de programas voltados ao controle de doenças transmissíveis e não transmissíveis, assim como as tarefas administrativas. Observa-se uma diferença marcada nas ações de saúde voltadas às mulheres, que no Chile são responsabilidade das obstetrizes e, na Costa Rica, responsabilidade exclusiva das enfermeiras obstetras. Em termos gerais, a atuação das enfermeiras é semelhante; contudo, o alcance de suas funções e sua capacidade de resolver problemas de saúde estão relacionados ao nível de organização da APS em cada país. 

Nos cinco países, assinala-se a necessidade de que as enfermeiras na APS trabalhem mais próximas da comunidade, com foco na prevenção de doenças e nos determinantes sociais da saúde. 

O grupo focal do México indicou que as enfermeiras recebem formação para trabalhar na APS, mas o mercado de trabalho não favorece essa atuação. Na Colômbia, o sistema de saúde não estimula essa formação e há poucos cenários de prática no primeiro nível de atenção, apesar do potencial desses profissionais para atuar na comunidade. Por outro lado, a Costa Rica aponta a fragilidade da formação para a atenção primária, enquanto o Chile indica a necessidade de discutir o papel das enfermeiras nesse nível de atenção.

Além desses elementos, foram identificados fatores que limitam o trabalho das enfermeiras, como o excesso de atividades administrativas, que dificultam as ações voltadas ao cuidado, e a insuficiência da formação de graduação no âmbito de atuação que a APS requer. 

Apesar de tais limitações na APS nesses países, foram assinaladas algumas potencialidades. No caso do Brasil, destaca-se o papel de liderança que as enfermeiras exercem na equipe interprofissional, possivelmente o âmbito em que essas profissionais têm maior protagonismo e autonomia e, em algumas regiões do país, podem contar com protocolos que lhes permitem ampliar o alcance de sua prática clínica.

No Chile, a APS é um espaço onde historicamente se tem reconhecido o trabalho das enfermeiras, que ocuparam cargos diretivos nas instituições de saúde e foram responsáveis por programas relevantes, como o de imunizações. Ao mesmo tempo, é o nível de atenção que em maior medida favorece o trabalho interprofissional. 

Na Colômbia, propõe-se um modelo de atenção com maior ênfase na prevenção e, a partir disso, surgiu a proposta de que as enfermeiras trabalhem mais estreitamente com a comunidade. Reconhece-se que o território e as comunidades oferecem oportunidades de liderança para a Enfermagem para enfrentar novos problemas de saúde e transformar a atenção. 

Na Costa Rica, as enfermeiras desempenham um papel essencial no primeiro contato dos usuários com o sistema de saúde. Embora se assinale que algumas ações de cuidado são delegadas a auxiliares de enfermagem, destaca-se a função gestora das enfermeiras, bem como a necessidade de ampliar sua liderança na comunidade. Esse trabalho comunitário também é destacado no México, onde se observam bons resultados quando é realizado por profissionais de enfermagem e se considera um elemento transformador da realidade sanitária e do desenvolvimento comunitário.

### Formação em nível de graduação e pós-graduação

A formação das enfermeiras nos cinco países é de nível universitário; embora existam aspectos semelhantes, também se observam certas particularidades, como indicado na Figura 1.

**Figura 1:** Características da formação de graduação em enfermagem no Brasil, Chile, Colômbia, Costa Rica e México, 2023

**País**	**Características**
**Brasil**	Diretrizes curriculares nacionais adaptadas às necessidades regionais e aos perfis epidemiológicos^11^. Duração de 4 a 6 anos, com um mínimo de 3.600 horas^12^. As atividades teóricas e práticas são desenvolvidas ao longo de toda a formação^11^. Estágio em condições reais de trabalho no último ano (com uma distribuição equitativa da carga de trabalho entre as áreas hospitalar e de Atenção Primária de Saúde)^11^. Dos cursos, 11,2% são ofertados em instituições públicas e 88,8% em instituições privadas^13^.
**Chile**	Duração de 5 anos (10 semestres)^14^. O conteúdo da formação é definido pelas universidades com o objetivo de cumprir as quatro funções das enfermeiras: cuidado, educação, pesquisa e trabalho na comunidade. Os currículos de graduação incluem disciplinas fundamentais, tais como: 1) Humanidades (antropologia, filosofia, ética, psicologia, entre outras); 2) Ciências básicas (morfologia, fisiologia, fisiopatologia, farmacologia, bioquímica, entre outras); 3) Ciências disciplinares (modelos e teorias de enfermagem, processo de cuidado de enfermagem, cuidado de enfermagem nas diferentes etapas do ciclo vital, gestão e administração em enfermagem, entre outras)^15^. Não existem números exatos de instituições públicas e privadas.
**Colômbia**	Duração de 8 a 10 semestres^16^. A formação se organiza em três componentes: 1) Ciências sociais e ciências naturais; 2) Área disciplinar específica da prática de enfermagem (70 a 80%); 3) Interface com outras áreas do conhecimento. Um ano de trabalho social ao final do curso (com escolha de diferentes áreas). Sendo 37% em instituições públicas e 63% em instituições privadas^16^.
**Costa Rica**	Duração de 4 a 5 anos. Não existe um currículo unificado: a formação varia consideravelmente entre universidades, com maior ou menor ênfase no cuidado, na educação ou na atenção primária. O país conta com órgãos reguladores da educação superior. A formação se estrutura em uma fase teórica seguida de uma fase prática supervisionada. O país conta com uma universidade pública e sete privadas.
**México**	Dois tipos de cursos: 1) Curso de nivelamento: dirigido a quem possui formação técnica; com duração entre um e dois anos, segundo o programa; 2) Licenciatura de quatro anos. Ao final da licenciatura, exige-se um ano adicional de trabalho social na atenção primária à saúde. A formação é regida pela Lei de Educação Superior (licenciatura e pós-graduação). Não se dispõe do número exato de instituições públicas e privadas.

No Brasil existe preocupação com o crescimento exponencial do número de universidades privadas e com a adoção da educação a distância, nem sempre com a qualidade formativa que é exigida pelos profissionais e pela população. Além disso, preocupa a fragilidade do processo de avaliação para a criação e o funcionamento contínuo dos cursos^9^.

No Chile, a profissão de enfermeira está regulamentada por lei desde 1997^10^. O curso de Enfermagem é de nível universitário, mas não é reconhecido como tal na lei de educação. Um conjunto de normas sobre a acreditação e o licenciamento dos cursos de Enfermagem está em tramitação na *Comisión Nacional de Acreditación*. Além disso, promover uma formação menos centrada no paradigma biomédico continua sendo um desafio para muitas universidades. 

A Colômbia conta com uma formação clínica sólida, devido, entre outros fatores, aos cenários de prática propiciados pelo sistema, nos quais os postos de trabalho são majoritariamente hospitalares. O sistema de saúde, centrado na atenção especializada e na subespecialidade, acaba por configurar os currículos, a formação e o marco de competências e habilidades.

Na Costa Rica, a variedade de currículos continua propiciando que, em muitas instituições, predomine o paradigma biomédico. Essa dificuldade na formação repercutiu no trabalho das enfermeiras do sistema de saúde costarriquenho.

Assim como em outros países, o modelo de formação biomédico constitui um aspecto limitante no México para a formação das enfermeiras, o que repercute no enfoque interprofissional.

A formação de pós-graduação é mais diversa que a de graduação, tanto no desenvolvimento dos programas como no papel desempenhado pelas especializações para a integração das enfermeiras no mercado de trabalho. A Figura 2 apresenta um panorama da formação de pós-graduação nesses países.

**Figura 2:** Tipos e características da formação de pós-graduação no Brasil, Chile, Colômbia, Costa Rica e México, 2023

Cursos	Brasil	Chile	Colômbia	Costa Rica	México
**Especialização**	Mínimo de 360 horas; cursos em diversas áreas	1 ano; enfoque clínico pós-títulos ou cursos de especialização	46 áreas 1 ano/1 ano e meio	Não há	Cursos em diversas áreas do cuidado de enfermagem
**Especialização em formato de residência (modalidade teórico-prática)**	2 anos; mínimo de 5.760 horas	Não há	Não há	Não há	Não há
**Mestrado acadêmico**	54	Número não identificado	Número não identificado	Número não identificado	Número não identificado
**Mestrado profissional ou de aprofundamento**	24	Número total de mestrados profissionais não identificado; dois mestrados para EPA*.	Cifra no identificada	Cifra no identificada	Número total de mestrados profissionais não identificado; um mestrado para EPA*.
**Doutorado acadêmico**	41	2	3	Não há	Número não identificado
**Doutorado profissional**	2	Não há	Não há	Não há	Não há

*EPA = Enfermeira de prática avançada

O Brasil tem o maior número de programas de mestrado e doutorado. Os cursos são padronizados e avaliados pela Coordenação de Aperfeiçoamento de Pessoal de Nível Superior (CAPES) e seguem as mesmas regras para todas as áreas do conhecimento. A maioria dos cursos de pós-graduação é ofertada em universidades públicas e é gratuita. Por intermédio da CAPES e de outros órgãos de fomento, é possível obter bolsas para essa formação. O país conta com programas de especialização em formato de residências, específicas de enfermagem (uniprofissionais) ou multiprofissionais, com bolsas. Além disso, existem especializações em diversas áreas da enfermagem e áreas afins. Essa é a modalidade de pós-graduação mais acessível, mas também a menos regulada. Como resultado disso, e devido à expansão da educação privada no país, essa modalidade se intensificou, com a criação de um grande número de cursos, sobretudo na modalidade a distância. 

O Chile conta com mestrados em enfermagem orientados à pesquisa e mestrados em ciências da saúde ou de caráter interprofissional aos quais as enfermeiras podem ter acesso. Mais recentemente, foi criado um mestrado centrado na prática clínica, que confere um título acadêmico e está voltado para a formação da EPA na área de oncologia. No que se refere aos doutorados, existem dois programas em enfermagem. O reconhecimento de especialidades depende da regulamentação do Ministério da Saúde para a área da saúde, que não inclui as especialidades de enfermagem. Nesse caso, não se amplia o escopo de prática e as enfermeiras continuam exercendo as mesmas funções, apenas com maior conhecimento e, consequentemente, sem diferenças salariais.

A Colômbia conta com programas de mestrado e doutorado; essa formação é considerada sólida, mas existe a possibilidade de ampliá-la, incluindo a prática em cenários de saúde pública. Em relação às especialidades, foi apontado que as enfermeiras enfrentam dificuldades para realizar estudos de pós-graduação devido ao custo e aos obstáculos para conciliar vida laboral e familiar; além disso, existe desinteresse em razão da falta de reconhecimento da especialidade.

A Costa Rica conta com programas de mestrado em enfermagem em instituições públicas e privadas. O *Colegio de Enfermeras de Costa Rica* dispõe de um regulamento para o registro e reconhecimento dos cursos de pós-graduação, que abrange tanto os cursos oferecidos no país quanto os realizados no exterior. No país não existe um programa de doutorado em enfermagem, mas há cursos em áreas afins, como educação, cultura e outras áreas cursadas pelas enfermeiras. Outro desafio é o reconhecimento governamental dos cursos de pós-graduação, que reconhece apenas os de saúde mental e psiquiatria, e o de ginecologia e obstetrícia; no entanto, o título nem sempre se traduz em salários mais altos e a formação implica um custo.

No México são oferecidos programas de mestrado e doutorado em universidades públicas e privadas; além disso, algumas especialidades foram convertidas em mestrados, mas ainda são oferecidos cursos que conferem o título de especialista mediante certificação profissional. A pós-graduação nem sempre conduz a melhores salários, mas o curso de especialização é um requisito para o acesso a alguns postos de trabalho.

### Regulação do escopo de prática

Em todos os países, a legislação é aplicada em nível nacional; no México, existe uma legislação nacional geral, embora o alcance do exercício profissional possa variar entre os subsistemas sanitários e suas instituições, assim como entre os estados da federação. O procedimento de registro profissional e os instrumentos normativos que regulam a prática da enfermagem são apresentados na Figura 3.

**Figura 3:** Registro profissional e instrumentos normativos da regulação do exercício da enfermagem no Brasil, Chile, Colômbia, Costa Rica e México, 2023

**País**	**Registro profissional e instrumentos normativos**
**Brasil**	Registro profissional: Conselho Federal de Enfermagem Instrumentos normativos: Lei do exercício profissional, n.º 7.489/1986^17^, Código de deontologia profissional^18^; além de resoluções, decisões e pareceres normativos do Conselho Federal e do Sistema de Conselhos Regionais de Enfermagem^19^, assim como portarias, decretos e outros documentos normativos do Ministério da Saúde e secretarias de saúde.
**Chile**	Registro profissional: Colégio de Enfermeiras do Chile^20^ Instrumentos normativos: Lei 19.536/1997 - reconhece o papel da enfermeira no Código Sanitário chileno; Lei 19.937/2004 - insere a “gestão do cuidado” na estrutura dos Serviços de Saúde dependentes do Estado, entendendo-a como o papel da enfermeira^21^ ^-^ ^22^; e o Código de Ética - 2008^20^.
**Colômbia**	Registro profissional: Registro Único Nacional de Enfermagem na *Asociación Nacional de Enfermeras de Colombia* e, em nível nacional, a *Organización Colegial de Enfermería* expede a Carteira Nacional de Identifica*ção* com base na inscrição no *Registro Único Nacional del Talento Humano en Salud*. Instrumentos normativos: Lei de regulamentação profissional - Lei 266/1996^23^; Lei 911/2004 - Tribunal Nacional de Ética em Enfermagem^24^.
**Costa Rica**	Registro profissional: Colégio de Enfermeiras da Costa Rica. Instrumentos normativos: Lei Orgânica do Colégio de Enfermeiras (Lei 2.343/1959) e Lei 7.085/87 - estabelece os diferentes níveis de enfermeiras que existem nas instituições de saúde, dependendo da complexidade dos departamentos ou serviços de enfermagem e do volume de trabalho.
**México**	O registro e a licença de profissionais são realizados junto ao Governo mexicano. No ano 2000 instituiu-se a certificação de enfermagem (um procedimento voluntário que não substitui a licença, e a falta de certificação não implica a perda da licença). Existem duas organizações com autorização oficial para a certificação: o *Consejo Mexicano para la Certificación de Enfermería* e o *Colegio Mexicano de Licenciados en Enfermería* ^25^. Instrumentos normativos: *Ley General de Profesiones* (1945) e existem as *Normas Oficiales Mexicanas* (NOM) que estabelecem os espaços de prática da enfermagem no território nacional^25^.

Outro elemento debatido foi o desempenho de enfermeiras graduadas como auxiliares ou técnicas de enfermagem, prática presente na Costa Rica e no México, enquanto no Brasil não é possível que uma enfermeira atue como auxiliar ou técnica de enfermagem, a menos que tenha concluído o curso de auxiliar ou técnica e esteja registrada nesse nível.

### Iniciativas para ampliar o escopo de prática das enfermeiras nos serviços de atenção primária à saúde

O contexto de ampliação do escopo de prática das enfermeiras nos países analisados é heterogêneo e depende da fase de desenvolvimento da prática e da formação em enfermagem em cada país, assim como das estratégias de regulação da prática profissional, das características dos sistemas de saúde e das disposições e normas das instituições de saúde. A seguir, apresentam-se os principais aspectos por país.

#### Brasil

No Brasil, o Conselho Federal de Enfermagem (COFEN) e a Associação Brasileira de Enfermagem criaram grupos de trabalho para promover debates sobre o tema e elaborar documentos. Em 2023, o Ministério da Saúde criou um grupo de trabalho integrado por diferentes associações e especialidades da enfermagem, que assumiu um papel de liderança nos debates sobre a prática em enfermagem e as possibilidades de ampliar o papel das enfermeiras. A pesquisa sobre o tema também tem sido fomentada e diversos artigos foram publicados em diferentes revistas científicas.

Desde 2021, o COFEN tem buscado o intercâmbio de experiências internacionais para apoiar os processos de ampliação do escopo de prática. A organização estabeleceu alianças com a CAPES por meio do apoio financeiro aos mestrados profissionais já existentes no Brasil e impulsionou o desenvolvimento de propostas de mestrados em EPA. 

A lei do exercício profissional garante às enfermeiras a possibilidade de ampliar seu escopo de prática em programas de saúde pública, mediante protocolos adequados^17^. Considerando que o país adota o modelo de autorregulação da profissão pelos conselhos profissionais, isso favorece a atualização de resoluções e atos normativos que se consideram necessários para respaldar o exercício profissional. 

Ao longo dos anos, observou-se uma ampliação da função da enfermeira em diversas áreas, por meio de várias especialidades, como a neonatologia, a obstetrícia na atenção secundária e o apoio diagnóstico por meio de ultrassonografia. Assim como na APS, a função dessas profissionais foi ampliada a partir do desenvolvimento de protocolos clínicos em diversas áreas, o que permite considerar que práticas avançadas já estão sendo desenvolvidas.

Apesar desse cenário, ainda existem desafios para a ampliação do escopo de prática: entre eles, a formação de especialistas, a regulamentação, o mercado de trabalho e a aceitação das EPA por parte de outros profissionais, dos serviços e provedores de saúde e da população.

Entre os elementos que devem ser analisados figuram se a EPA constitui outra categoria profissional e como se relacionaria com as enfermeiras, que já contam com um escopo de atuação ampliado no Brasil, e a adequação do modelo formativo, dado que existem opiniões divergentes sobre a formação em nível de mestrado ou de residência. 

#### Chile

No Chile, a implementação da EPA foi realizada por meio de debates com atores internacionais e iniciativas que utilizaram o processo participativo para a enfermagem de prática avançada, centrado no paciente e baseado em evidências (PEPPA, em inglês)^26^, que desenvolve o programa a partir das necessidades do país e identifica os interlocutores para sua inserção nos espaços clínicos.

A *Universidad Católica de Chile* assumiu o desafio de criar um programa de formação em EPA e, desde o início, considerou-se necessário abordar os aspectos políticos para formar criticamente as enfermeiras. No entanto, as tentativas de ampliar a função na APS encontraram resistência por parte dos médicos de família e comunidade. Ao mesmo tempo, geraram-se as condições para ampliar a função das enfermeiras em oncologia a partir do *Plan Nacional del Cáncer* 2018-2028, que contempla a criação de um programa de pós-graduação para profissionais não médicos, orientado a ampliar o escopo de prática^27^. Foram então criados dois programas de mestrado de EPA nessa área.

A EPA em oncologia foi facilitada no ambiente clínico, uma vez que as enfermeiras já estavam inseridas e desempenhavam funções estabelecidas, de modo que continuaram nos mesmos cargos e funções, porém com uma formação sólida, o que resultou em melhorias na atenção aos pacientes. No entanto, a remuneração constitui o principal desafio, pois não foram efetuados aumentos salariais nesse novo papel nem foram definidos cargos ou funções específicas.

Existem iniciativas por parte do Ministério da Saúde para o reconhecimento de especialidades; entre elas, a EPA. Contudo, no país persistem conflitos em torno da regulação do exercício profissional, que exigem um debate mais amplo sobre a formação e a prática em saúde.

Apesar desses desafios, as perspectivas de ampliar o escopo de prática continuam sendo promissoras e tem havido um amplo debate a esse respeito. Propõe-se como premissa que é fundamental considerar o enfoque da APS.

#### Colômbia

Existem diferentes iniciativas para desenvolver o papel da EPA nos diferentes níveis de atenção, que contam com o apoio do Ministério da Saúde. Foram realizados sete encontros sobre a enfermagem de prática avançada, com a participação de pessoas que apoiaram experiências em outros países.

Da mesma forma, a reforma do sistema de saúde está orientada ao fortalecimento das ações preventivas e buscou-se estabelecer o marco normativo que inclua o financiamento da formação em prática avançada. A prioridade é iniciar a formação nas universidades públicas e definir o perfil profissional da EPA na APS.

A posição do amplo grupo de formação avançada, integrado por diretores de programas de mestrado e doutorado e membros da diretoria da *Asociación Colombiana de Facultades y Escuelas de Enfermería*, sustenta que o nível de formação da EPA deve ser o de mestrado de aprofundamento.

Apesar das iniciativas, os desafios da implementação residem em vários aspectos, entre eles, a oferta e o acesso a cursos, acompanhados de uma norma do Ministério que permita o desenvolvimento da prática e apoie os estudos de pós-graduação.

Outra dificuldade é o baixo reconhecimento da formação de pós-graduação, que nem sempre implica reconhecimento por parte dos pares e da sociedade, nem se traduz em níveis salariais superiores. Existem outros aspectos que favorecem a formação em nível de mestrado, como a oferta de bolsas que incentivam a capacitação. Também se buscou um incentivo econômico por meio do subsídio educacional mensal previsto na *Ley de Residentes*, atualmente exclusiva para médicos. As residências, como parte da formação de pós-graduação em enfermagem, favoreceriam a melhoria da prática clínica mediante um processo de imersão e com salários que permitam dedicação a essa formação.

De forma mais imediata, avançou-se na definição do perfil com o objetivo de orientar a implementação da EPA. Também houve avanços nos processos curriculares e organizativos dos cursos.

#### Costa Rica

Em 2017, o Colégio de Enfermeiras da Costa Rica realizou a primeira reunião para iniciar o processo de análise e definição da linha de trabalho para o desenvolvimento da EPA. Em 2019 foi realizada a primeira oficina nacional de EPA, por meio de um grupo de trabalho intersetorial que envolveu o setor acadêmico e buscou definir ações de curto e médio prazo.

Nas universidades existem iniciativas para analisar a possibilidade de oferecer formação em prática avançada com grupos de enfermeiras líderes na América Central. Em relação à prática de enfermagem, assinala-se a necessidade de superar a escassez de enfermeiras e a infraestrutura deficiente, bem como de consolidar a liderança das enfermeiras.

A Costa Rica também apresenta uma alta porcentagem de enfermeiras graduadas subcontratadas em funções aquém de seu nível de formação. Nesse processo de debate, foram identificados temas que requerem maior aprofundamento no país, como a regulação do exercício profissional.

É necessário pensar em um perfil de profissional com funções avançadas que, de fato, possa responder às necessidades de saúde da população; o que implica necessariamente o processo de formação e como serão delineadas as propostas de prática avançada. Portanto, para alcançar mudanças estruturais, é necessário contar com um perfil profissional de enfermagem não apenas qualificado do ponto de vista assistencial e da gestão do sistema, mas também com competências políticas que posicionem as enfermeiras nas mesas de decisão e lhes permitam influenciar a construção de políticas mais amplas.

#### México

Em 2019 constituiu-se um grupo de trabalho sobre a ampliação do escopo de prática das enfermeiras, liderado pela OPAS, pela *Universidad Autónoma de México*, pela *Universidad Autónoma de Xochimilco* e pela *Secretaría de Salud*, com a participação do Colégio de Enfermeiras, da *Comisión Permanente de Enfermería da Secretaría de Salud* e das universidades.

Entre os produtos está o desenvolvimento de um marco normativo mexicano de competências de enfermagem, com seis domínios e 68 competências para a lista ampliada de funções das enfermeiras com experiência na APS. A estratégia do México para potencializar o trabalho das enfermeiras tem duas vertentes: 1) ampliar as funções daquelas que trabalham há dois anos ou mais na APS, na medida em que a normativa vigente o permita; 2) estabelecer o papel da EPA no país, o que inclui mudanças normativas e formação em nível de mestrado.

A *Secretaría de Salud* assumiu um papel central em todo o processo e prepara-se para lançar experiências-piloto em vários estados da República Mexicana, nas quais essas enfermeiras de prática avançada serão alocadas em centros de saúde onde poderão desempenhar funções ampliadas. Para isso, foram formuladas propostas de programas de formação de EPA baseadas nas recomendações internacionais para mestrados e com um enfoque de saúde pública. O primeiro programa de mestrado foi realizado entre 2021 e 2022.

Cabe destacar que, o debate em torno da ampliação da função das enfermeiras no México também se desenvolveu por vias paralelas, pois envolveu outros atores fora do sistema de saúde.

Ainda permanecem desafios a serem superados nesse processo, e o principal deles é a desvalorização das enfermeiras graduadas. As especialidades não são garantia de melhores salários, motivo pelo qual causa preocupação a exigência de um mestrado para a EPA. Também é necessário avançar no âmbito regulatório, incluindo a consideração das diferenças no alcance das práticas já regulamentadas entre os distintos estados.

## Discussão

A formação e a prática da enfermagem são heterogêneas nos contextos analisados. A configuração do escopo de prática nos países analisados tem sido definida em função de suas realidades específicas. Apesar das semelhanças no papel das enfermeiras, o alcance de suas ações, seu reconhecimento e sua influência continuam sendo díspares.

A concepção de prática avançada e as motivações e interesses para implementá-la também variam entre países^28^. Do mesmo modo, uma análise da situação da EPA na Europa revela importantes variações na regulação, na formação e no escopo de prática, com diferentes níveis de competências e autonomia na atenção à saúde^29^ ^-^ ^30^.

Tem sido apontada a subutilização do potencial das enfermeiras, de sua formação e capacidade para atender às necessidades da população e contribuir para a gestão e a formulação de políticas de saúde. Essa subutilização se manifesta tanto na escassez de postos de trabalho quanto na falta de reconhecimento dessas profissionais para ocupar cargos estratégicos.

Por se tratar de uma função incipiente nos países objeto deste estudo, identifica-se uma fragilidade em termos de regulação, sendo necessário estabelecer um marco normativo que reconheça formalmente a função, defina os critérios de licença e de renovação do registro, como demonstra a experiência de países nos quais a função já está consolidada^31^.

Nesse contexto, é fundamental estabelecer as bases para definir as normas da prática e defender regulamentos específicos^32^. Os processos regulatórios devem ser revisados e atualizados com base nas necessidades dos sistemas locais de saúde e na criação de espaços laborais com proteção social para os trabalhadores.

Identificar e enfrentar as barreiras regulatórias é essencial para o pleno desempenho da prática da EPA e para ampliar suas contribuições potenciais à inovação e à transformação dos modelos de atenção^33^.

Entretanto, os achados do estudo indicam que prevalece uma regulação deficiente^34^, o que pode fazer com que essa titulação não seja reconhecida de imediato e, além disso, implica baixa inserção no mercado, falta de definição do papel, diferenciação salarial e desestímulos para continuar a capacitação, agravados ainda pelo fato de que, em alguns países, o investimento econômico necessário para acessar os programas de pós-graduação é elevado.

É importante garantir que as enfermeiras possam desempenhar plenamente suas funções, em conformidade com seu marco normativo, para que o trabalho dessas profissionais tenha impacto significativo. É necessário ampliar o número de postos de trabalho, principalmente na APS, garantir condições de trabalho dignas e revisar os processos de trabalho para reposicionar o papel das enfermeiras no contexto das equipes interprofissionais de saúde.

Investir na enfermagem e reconhecer sua função implica reduzir as desigualdades de gênero, dado que se trata de uma categoria profissional majoritariamente feminina, cujas integrantes enfrentam diferenças nas condições de trabalho, nas oportunidades de ascensão e liderança e na remuneração^35^.

No que diz respeito ao desenvolvimento de capacidades, a ampliação do escopo de prática requer uma formação coerente e de qualidade, que proporcione aos profissionais conhecimentos amplos e pensamento crítico sobre as condições de saúde.

Os resultados apresentados neste estudo indicam distintas possibilidades de programas de formação, com ênfase nos mestrados ou nos programas de residência. A experiência internacional aponta para a adoção de programas formais de formação, sendo os mestrados os mais frequentes^31^.

Recomenda-se ampliar os programas de formação mediante apoio financeiro público que permita maior dedicação e reduza a preocupação com a renda familiar.

Os países analisados neste estudo apresentam diferenças quanto à oferta de programas de formação. A oferta e a capacidade formativa em especialização, mestrado e doutorado devem ser otimizadas para a formação da EPA; no entanto, é necessário identificar e definir as competências requeridas para essas profissionais, o que facilitará a compreensão, a visibilidade e a implementação da EPA nos serviços de saúde^36^.

Também é importante discutir a função desse profissional a partir da educação e da prática em equipes interprofissionais^37^ ^-^ ^38^. A ampliação do papel das enfermeiras, a combinação de tarefas e o desenvolvimento de equipes interprofissionais são estratégias que podem ser empregadas para enfrentar a escassez de recursos humanos para a saúde e obter resultados positivos na melhoria da atenção à saúde da população e na satisfação dos profissionais e dos usuários, especialmente na APS^37^. As evidências indicam que a integração da EPA contribui para melhorar os resultados assistenciais, ampliar o acesso, reduzir os custos sanitários, melhorar a qualidade da atenção e aumentar o bem-estar dos pacientes^39^ ^-^ ^40^.

No entanto, é necessário promover um entendimento compartilhado entre as equipes de saúde, reconhecendo o papel e as contribuições da EPA, sua experiência e autonomia na atenção à saúde^41^.

A implementação efetiva e oportuna da EPA deve basear-se nas necessidades de saúde e não em imperativos da força de trabalho nem dos profissionais. Embora o processo de implementação possa ser prolongado, a experiência de outros países nos quais a função foi implementada revela a importância de um debate amplo e de uma colaboração efetiva entre os responsáveis políticos, os educadores, os empregadores e as associações profissionais para garantir que todas as iniciativas se alinhem aos objetivos durante sua implementação^30^ ^,^ ^42^.

Do mesmo modo, é necessário estimular a participação dessas profissionais na formulação de políticas e nos espaços de tomada de decisão. Considera-se desejável fornecer mais informações à sociedade sobre seu trabalho, com ênfase na evidência científica de seus resultados.

Uma limitação deste estudo reside na natureza dos dados, baseados nas percepções e experiências de participantes selecionados. No entanto, a inclusão de diferentes atores-chave em cada país e a triangulação com dados secundários reforçam a robustez e a credibilidade dos achados. Apesar dessa limitação, o estudo traz contribuições relevantes ao apresentar uma análise comparativa de diversos contextos nacionais, o que permite identificar fatores facilitadores e desafios comuns para a ampliação do escopo de prática da enfermagem na Região das Américas.

## Conclusão

A implementação da função de enfermeira de prática avançada constitui uma oportunidade real para que os países ampliem o acesso e a cobertura de saúde, especialmente na atenção primária à saúde. Há interesse e iniciativas para ampliar a função das enfermeiras na América Latina; no entanto, ainda há um caminho a percorrer em termos de formação, escopo de prática, regulação e mercado de trabalho.

O setor acadêmico desempenhou um papel importante ao fomentar a implementação da EPA por meio do desenvolvimento de programas de formação. No entanto, a experiência dos países latino-americanos e de outros países nos quais a EPA está consolidada evidencia o papel dos grupos de trabalho e das ações e programas intersetoriais, com ampla participação de diversos atores no diálogo político para a implementação efetiva e oportuna dessa função.

## Data Availability

Os conjuntos de dados relacionados a este artigo estarão disponíveis mediante solicitação ao autor correspondente.

# Translation: Ampliación del ámbito de práctica de enfermeras: posibilidades en cinco países de América Latina^*^

**DOI:** 10.1590/1518-8345.8037.4818

**Authors:** Bruna Moreno-Dias, Livia Angeli-Silva, Clara Aleida Prada-Sanabria, Tiago Parada Costa Silva, Silvia Helena de Bortoli Cassiani, Eduardo Benjamin Puertas-Donoso

**Affiliations:** 1 Organización Panamericana de la Salud, Departamento de Sistemas y Servicios de Salud, Washington, DC, Estados Unidos de América.; 2 Universidade Federal da Bahia, Escola de Enfermagem, Salvador, BA, Brasil.; 3 Universidade Estadual de Feira de Santana, Departamento de Saúde, Feira de Santana, BA, Brasil.

**Keywords:** Enfermería de Práctica Avanzada, Enfermería de Atención Primaria, Rol de la Enfermera, Educación de Postgrado en Enfermería, Fuerza Laboral en Salud, Sistemas de Salud.

## Abstract

**Objetivo:**

analizar, desde una perspectiva comparada, la formación y la regulación del trabajo de las enfermeras y las propuestas para la implementación de la función de las enfermeras de práctica avanzada en países seleccionados de América Latina.

**Método:**

estudio descriptivo de casos comparados para comprender la formación y la regulación del trabajo de enfermería en cinco países: Brasil, Chile, Colombia, Costa Rica y México. Los datos se recopilaron en cinco grupos focales con informantes clave de cada país. El contenido se sistematizó en una matriz analítica que fue validada por los participantes.

**Resultados:**

los países se encuentran en diferentes fases de debate e implementación de la ampliación del ámbito de práctica. El primer nivel de atención se considera un espacio potencial para el desempeño de esta función. Asimismo, los programas de maestría constituyen una oportunidad de formación para estas profesionales. El intercambio de experiencias internacionales y los grupos de trabajo han sido iniciativas para debatir la función y considerar las características específicas del país en su proceso de implementación.

**Conclusión:**

hay interés e iniciativas para ampliar la función de las enfermeras en América Latina; sin embargo, aún queda camino por recorrer en términos de formación, ámbito de práctica, regulación y mercado laboral.

## Destacados

**(1)** Hay una infrautilización de las contribuciones y las competencias de las enfermeras. **(2)** El marco regulatorio debe actualizarse desde una perspectiva interprofesional. **(3)** Grupos de trabajo e intercambio de experiencias han sido iniciativas para debatir EPA. **(4)** El desarrollo de programas de formación en algunos países ha fomentado la implantación. **(5)** Un amplio debate con diferentes actores y sectores es esencial para hacer avanzar la EPA.

## Introducción

Las enfermeras constituyen un importante contingente en los sistemas de salud de los países de la Región de las Américas y son reconocidas como profesionales esenciales para el acceso y la cobertura universal de la salud^1^.

Las desigualdades en la distribución de los profesionales, asociadas a la necesidad de aumentar el acceso de la población a servicios de salud y a profesionales de calidad, han contribuido al desarrollo, en algunos países, de profesionales con funciones ampliadas, como la enfermera de práctica avanzada (EPA), con el fin de mejorar el acceso a los servicios de salud, garantizar la cobertura universal, promover la sostenibilidad de los sistemas de salud y satisfacer las necesidades de salud globales^1^ ^-^ ^3^.

La Organización Panamericana de la Salud (OPS) considera las EPA como profesionales con formación de posgrado que, integradas en equipos interprofesionales, contribuyen a la gestión de los cuidados de los pacientes o usuarios con enfermedades agudas leves y trastornos crónicos diagnosticados, según protocolos o guías clínicas. El ejercicio profesional ampliado se caracteriza por un grado de autonomía en la toma de decisiones, que incluye el diagnóstico y el tratamiento de los trastornos de los pacientes^3^.

En algunos países, sobre todo en aquellos con economías más sólidas y sistemas de salud con mayor cobertura poblacional, se ha valorado positivamente la ampliación de las funciones de las enfermeras, especialmente en la continuidad de la atención, en la reducción de costos y en la satisfacción de los usuarios^4^ ^-^ ^6^.

En las Américas, las experiencias de Canadá y Estados Unidos están consolidadas. Por su parte, América Latina se encuentra en una fase incipiente y diversificada en cuanto a la comprensión, la formación y la regulación de las EPA, a pesar de las iniciativas dirigidas a su desarrollo^2^ ^-^ ^3^.

El debate sobre el marco regulatorio, el desarrollo de competencias y las condiciones de trabajo de los profesionales con ámbito de práctica ampliado representa una oportunidad para los países de la Región de las Américas, como se destaca en la “Política sobre el personal de salud 2030: fortalecimiento de los recursos humanos para la salud a fin de lograr sistemas de salud resilientes” de la OPS^7^.

La ampliación del ámbito de la práctica depende de la configuración de la enfermería en los países, por lo que, el conocimiento de las diferentes realidades es fundamental para avanzar en las políticas. La comprensión de las semejanzas y las diferencias de la enfermería y de su papel en los diferentes sistemas de salud de los países de América Latina contribuirán a señalar el alcance de la práctica que mejor responda a las necesidades de salud de cada país.

Por tanto, este artículo tiene como objetivo analizar, desde una perspectiva comparada, la formación y la regulación del trabajo de las enfermeras y las propuestas para la implementación del rol de las enfermeras de práctica avanzada en países seleccionados de América Latina.

## Método

### Tipo de estudio 

Estudio descriptivo de caso comparado^8^ para comprender la formación y la regulación del trabajo de enfermería.

### Escenario

Se seleccionaron cinco países de América Latina: Brasil, Chile, Colombia, Costa Rica y México, por considerarlos escenarios favorables para la implementación de la enfermería de práctica avanzada.

### Participantes

Se consideraron elegibles para el estudio informantes clave representantes de tres segmentos: asociaciones y colegios de enfermería; universidades o ámbitos de investigación especializados en el tema; y ministerios de salud, responsables de las políticas relativas al personal de salud. La composición de cada grupo tuvo en cuenta la presencia de al menos un representante de cada sector. Se identificó a los participantes con el apoyo de las oficinas de la OPS en los países incluidos en el estudio, así como por intermedio de la coordinación con asociaciones profesionales y ministerios de salud, mediante el envío de memorandos oficiales. Tras esta identificación, los posibles participantes recibieron, por correo electrónico, información detallada sobre la investigación y una invitación formal a participar. Se identificó a un total de 65 potenciales participantes, de los cuales participaron 39 (4 de Brasil, 14 de Chile, 6 de Colombia, 7 de Costa Rica y 8 de México). Se excluyó a las personas que, pese a haber sido nombradas, no respondieron a la invitación.

### Recolección de datos

Los datos se recopilaron mediante cinco grupos focales, uno por cada país, realizados entre junio y julio de 2023. Los grupos focales fueron moderados por dos investigadoras expertas en la temática y con experiencia en este tipo de estudios, quienes utilizaron una guía para la moderación del grupo de discusión y las siguientes preguntas disparadoras:

¿Cómo es la formación de las enfermeras licenciadas en su país (a nivel de grado y de posgrado)?

¿Cuál es la función desempeñada por las enfermeras licenciadas en el sistema de salud de su país?

¿Cuáles son las actividades desarrolladas por las enfermeras licenciadas en los servicios del primer nivel de atención/Atención Primaria de Salud?

¿Existen iniciativas o debates sobre la ampliación del papel de las enfermeras de práctica avanzada en el primer nivel de atención / Atención Primaria de Salud en su país?

Si existen iniciativas o propuestas para ampliar el papel de las enfermeras, ¿qué formación se ha exigido? ¿Cuáles son las formas de regulación y cómo se produciría la inserción laboral?

Cada grupo focal tuvo una duración de aproximadamente dos horas y se realizó mediante una plataforma virtual. Todas las sesiones se grabaron y se transcribieron íntegramente. Además, se realizaron búsquedas de documentos relevantes en sitios web oficiales de las autoridades sanitarias de los países incluidos en el estudio, en bases de datos indexadas y en los documentos citados por los participantes de los grupos focales.

### Análisis de datos

El contenido de la transcripción de los grupos focales se sometió a un análisis temático. Los resultados se sistematizaron en una matriz temática con cuatro categorías: el papel de las enfermeras; formación a nivel de pregrado y posgrado; la regulación del ámbito de práctica; y la ampliación del ámbito de práctica. Una vez que el equipo de investigación completó la matriz, esta fue validada por los participantes del estudio en una reunión virtual celebrada en septiembre de 2023, con duración de una hora y la participación de 14 personas que habían participado previamente de los grupos focales de cada país: 2 de Brasil, 4 de Chile, 2 de Colombia, 3 de Costa Rica y 3 de México.

### Consideraciones éticas

El protocolo de investigación fue analizado y aprobado por el Comité de Revisión Ética de la Organización Panamericana de la Salud (PAHOERC) con el dictamen PAHOERC.0622.01. El consentimiento para participar en el estudio se registró mediante un formulario electrónico compartido con los participantes al inicio del grupo focal.

## Resultados

La muestra del estudio estuvo compuesta por 39 participantes, en su mayoría, mujeres (62%). Se observó una distribución relativamente equilibrada por origen institucional: 38% provenían de asociaciones, colegios o federaciones profesionales, 33% de ministerios de salud o de seguridad social y 28% de instituciones académicas. Esta composición permitió reunir diversas perspectivas técnicas, científicas, políticas y estratégicas, así como visiones complementarias sobre los procesos de formación, el ámbito de práctica, las políticas y la gobernanza de la fuerza laboral de enfermería.

En Brasil, las cuatro participantes fueron mujeres: dos representantes de asociaciones y consejos profesionales, una del Ministerio de Salud y una proveniente de una institución académica. 

En Chile, de los 14 participantes, siete fueron mujeres; seis procedían de instituciones académicas, cinco de colegios profesionales o federaciones y tres del Ministerio de Salud. 

En Colombia, las seis participantes fueron mujeres: tres de asociaciones profesionales, dos del Ministerio de Salud y una proveniente de una institución académica. 

En Costa Rica, de los siete participantes, cinco fueron hombres; cuatro provenían de los servicios públicos (uno del Ministerio de Salud y tres de la Caja Costarricense de Seguro Social), del colegio profesional y uno a una institución académica. 

En México, de los ocho participantes, cinco fueron mujeres; tres provenían del Ministerio de Salud, tres de colegios o federaciones profesionales y dos de instituciones académicas.

A continuación, se presentan los resultados obtenidos en los grupos focales por categorías temáticas. Es importante destacar que, siempre que se trate de información procedente del análisis documental, se incluye la referencia correspondiente.

### El papel de las enfermeras 

Los cinco países comparten el predominio de las enfermeras en los servicios asistenciales, y también desempeñan un papel administrativo relevante; además, ocupan diferentes funciones en la estructura organizativa del sistema. Sin embargo, la presencia en espacios de decisión y en posiciones estratégicas continúa siendo un desafío.

Además, existen retos para que las enfermeras sean reconocidas por su trabajo. La subcontratación de enfermeras para funciones por debajo de su nivel de formación fue un factor señalado en Colombia, Costa Rica y México. Esto impide a un número importante de profesionales desempeñar plenamente las funciones para las que están formadas y cualificadas, lo que podría contribuir a lograr un sistema de salud más eficaz.

En el marco de la Atención Primaria de Salud (APS), entre las actividades comunes que las enfermeras desarrollan figuran la inmunización, la coordinación de programas dirigidos al control de enfermedades transmisibles y no transmisibles, así como las tareas administrativas. Una diferencia marcada se observa en las acciones de salud dirigidas a las mujeres, las cuales en Chile son responsabilidad de las matronas y, en Costa Rica, responsabilidad exclusiva de las enfermeras obstétricas. En términos generales, la actuación de las enfermeras es similar; sin embargo, el alcance de sus funciones y su capacidad para resolver problemas de salud están relacionados con el nivel de organización de la APS en cada país. 

En los cinco países se señala la necesidad de que las enfermeras en la APS trabajen más cerca de la comunidad, con enfoque en la prevención de enfermedades y en los determinantes sociales de la salud. 

El grupo focal de México indicó que las enfermeras reciben formación para trabajar en la APS, pero el mercado laboral no la favorece. En Colombia, el sistema de salud no favorece esta formación y hay pocos escenarios de práctica en el primer nivel de atención, a pesar del potencial de estos profesionales para trabajar en la comunidad. Por otro lado, Costa Rica señala la fragilidad de la formación para la atención primaria, mientras que Chile señala la necesidad de discutir el papel de las enfermeras en este nivel de atención.

Además de estos elementos, se han identificado factores que limitan el trabajo de las enfermeras, como el exceso de actividades administrativas, que dificultan las acciones orientadas a los cuidados, y la insuficiencia de la formación de pregrado en el ámbito de actuación que requiere la APS. 

A pesar de tales limitaciones en la APS en estos países, se han señalado algunas potencialidades. En el caso de Brasil, se destaca el papel de liderazgo que ejercen las enfermeras en el equipo interprofesional, posiblemente el ámbito en el que estas profesionales tienen mayor protagonismo y autonomía y, en algunas regiones del país, pueden contar con protocolos que les permiten ampliar el alcance de su práctica clínica.

En Chile, la APS es un espacio donde históricamente se ha reconocido la labor de las enfermeras, quienes han ocupado cargos directivos en las instituciones de salud y han sido responsables de programas relevantes, como el de inmunizaciones. Al mismo tiempo, es el nivel de atención que en mayor medida favorece el trabajo interprofesional. 

En Colombia se plantea un modelo de atención con mayor énfasis en la prevención, y a partir de ello ha surgido la propuesta de que las enfermeras trabajen más estrechamente con la comunidad. Se reconoce que el territorio y las comunidades ofrecen oportunidades de liderazgo para la enfermería para abordar nuevos problemas de salud y transformar la atención. 

En Costa Rica, las enfermeras desempeñan un papel esencial en el primer contacto de los usuarios con el sistema de salud. Aunque se señale que algunas acciones de cuidado se delegan a auxiliares de enfermería, se destaca la función gestora de las enfermeras, así como la necesidad de ampliar su liderazgo en la comunidad. Este trabajo comunitario también se destaca en México, donde se observan buenos resultados cuando lo realizan profesionales de enfermería y se considera un elemento transformador de la realidad sanitaria y del desarrollo comunitario.

### Formación a nivel de pregrado y posgrado

La formación de las enfermeras en los cinco países es de nivel universitario; aunque existen aspectos similares, también se observan ciertas particularidades, como se indica en la Figura 1. 

**Figura 1:** Características de la formación de pregrado en enfermería en Brasil, Chile, Colombia, Costa Rica y México, 2023

**País**	**Características**
**Brasil**	Pautas curriculares nacionales adaptadas a las necesidades regionales y a los perfiles epidemiológicos^11^. Duración de 4 a 6 años, con un mínimo de 3.600 horas^12^. Las actividades teóricas y prácticas se desarrollan a lo largo de toda la formación^11^. Pasantía en condiciones reales de trabajo en el último año (con un reparto equitativo de la carga de trabajo entre las áreas hospitalaria y de Atención Primaria de Salud)^11^. El 11,2% de los cursos se imparten en instituciones públicas y el 88,8% en instituciones privadas^13^.
**Chile**	Duración de 5 años (10 semestres)^14^. El contenido de la formación está definido por las universidades con el fin de cumplir con las cuatro funciones de las enfermeras: cuidado, educación, investigación y trabajo en la comunidad. Los currículos de pregrado incluyen asignaturas materias fundamentales, tales como: 1) Humanidades (antropología, filosofía, ética, psicología, entre otras); 2) Ciencias básicas (morfología, fisiología, fisiopatología, farmacología, bioquímica, entre otras); 3) Ciencias disciplinares (modelos y teorías de enfermería, proceso de atención de enfermería, atención de enfermería en las diferentes etapas del ciclo vital, gestión y administración de enfermería, entre otras)^15^. No existen cifras exactas de instituciones públicas y privadas.
**Colombia**	Duración de 8 a 10 semestres^16^. La formación se organiza en tres componentes: 1) Ciencias sociales y ciencias naturales; 2) Área disciplinar específica de la práctica de enfermería (70 a 80%); 3) Interfaz con otras áreas del conocimiento. Un año de trabajo social al final del curso (con elección de diferentes áreas). El 37% en instituciones públicas y el 63% en instituciones privadas^16^.
**Costa Rica**	Duración de 4 a 5 años. No existe un plan de estudios unificado: la formación varía considerablemente entre universidades, con mayor o menor énfasis en los cuidados, la educación o la atención primaria. El país cuenta con organismos reguladores de la educación superior. La formación se estructura en una fase teórica seguida de una fase práctica supervisada. El país cuenta con una universidad pública y siete privadas.
**México**	Dos tipos de cursos: 1) Curso de nivelación: dirigido a quienes cuentan con formación técnica; con una duración de entre uno y dos años, según el programa; 2) Licenciatura de cuatro años. Al final de la licenciatura, se requiere un año adicional de trabajo social en la atención primaria de salud. La formación está regida por la Ley de Educación Superior (licenciatura y posgrado). No se dispone de la cifra exacta de instituciones públicas y privadas.

En Brasil existe preocupación por el crecimiento exponencial del número de universidades privadas y por la adopción de la educación a distancia, no siempre con la calidad formativa que requieren los profesionales y la población. Asimismo, preocupa la debilidad del proceso de evaluación para la creación y el funcionamiento continuo de los cursos^9^.

En Chile, la profesión de enfermera está regulada por ley desde 1997^10^. La carrera de enfermería es de nivel universitario, pero no está reconocida como tal en la ley de educación. Un conjunto de normas sobre la acreditación y licenciamiento de las carreras de enfermería se tramita en la Comisión Nacional de Acreditación. Además, impulsar una formación menos centrada en el paradigma biomédico sigue siendo un reto para muchas universidades. 

Colombia cuenta con una formación clínica sólida, debido, entre otros factores, a los escenarios de práctica propiciados por el sistema, en los que los puestos de trabajo son mayoritariamente hospitalarios. El sistema de salud, centrado en la atención especializada y la subespecialidad, termina por configurar los currículos, la formación y el marco de competencias y habilidades. 

En Costa Rica, la variedad de planes de estudio sigue propiciando que en muchas instituciones predomine el paradigma biomédico. Esa dificultad en la formación ha repercutido en la labor de las enfermeras del sistema de salud costarricense.

Al igual que en otros países, el modelo de formación biomédico constituye un aspecto limitante en México para la formación de las enfermeras, lo que repercute en el enfoque interprofesional.

La formación de posgrado es más diversa que la de pregrado, tanto en el desarrollo de los programas como en el papel que desempeñan las especializaciones para la integración de las enfermeras en el mercado laboral. La Figura 2 ofrece un panorama de la formación de posgrado en estos países.

**Figura 2:** Tipos y características de la formación de posgrado en Brasil, Chile, Colombia, Costa Rica y México, 2023

Cursos	Brasil	Chile	Colombia	Costa Rica	México
**Especialización**	Mínimo de 360 horas; cursos en diversas áreas	1 año; enfoque clínico postítulos o diplomados	46 áreas 1 año/1 año y medio	No hay	Cursos en diversas áreas de los cuidados de enfermería
**Especialización en forma de residencia (modalidad teórico-práctica)**	2 años; mínimo de 5.760 horas	No hay	No hay	No hay	No hay
**Maestría académica**	54	Cifra no identificada	Cifra no identificada	Cifra no identificada	Cifra no identificada
**Maestría profesional o profundización**	24	Número total de maestrías profesionales no identificado; dos maestrías para EPA*.	Cifra no identificada	Cifra no identificada	Número total de maestrías profesionales no identificado; una maestría para EPA*.
**Doctorado académico**	41	2	3	No hay	Cifra no identificada
**Doctorado profesional**	2	No hay	No hay	No hay	No hay

*EPA = Enfermera de práctica avanzada

Brasil tiene el mayor número de programas de maestría y doctorado. Los cursos están estandarizados y evaluados por la *Coordenação de Aperfeiçoamento de Pessoal de Nível Superior* (CAPES) y siguen las mismas reglas para todas las áreas de conocimiento. La mayoría de los cursos de posgrado se imparten en universidades públicas y son gratuitos. Por intermedio de la CAPES y otros organismos de financiación, es posible obtener subvenciones para esta formación. El país cuenta con programas de especialización en forma de residencias, específicas de enfermería (uniprofesionales) o multiprofesionales, con becas. Asimismo, existen especializaciones en diversas áreas de la enfermería y afines. Esta es la modalidad de posgrado más accesible, pero también la menos regulada. Como resultado de ello, y debido también a la expansión de la educación privada en el país, esta modalidad se ha intensificado, con la creación de un gran número de cursos, sobre todo en modalidad a distancia. 

Chile cuenta con maestrías en enfermería orientadas a la investigación y maestrías en ciencias de la salud o de carácter interprofesional a las que pueden acceder las enfermeras. Más recientemente, se creó un máster centrado en la práctica clínica, que confiere un título académico y está dirigido a la formación de la EPA en el área de oncología. En cuanto a los doctorados, existen dos programas en enfermería. El reconocimiento de especialidades depende de la normativa del Ministerio de Salud para el área de la salud, que no incluye las especialidades de enfermería. En este caso, no se amplía el ámbito de práctica y las enfermeras siguen realizando las mismas funciones, solo que con más conocimientos y, en consecuencia, sin diferencias salariales.

Colombia cuenta con programas de maestría y doctorado; esta formación se considera sólida, pero existe la posibilidad de ampliarla, incluyendo la práctica en escenarios de salud pública. En cuanto a las especialidades, se señaló que las enfermeras encuentran dificultades para realizar estudios de posgrado dado el costo y los obstáculos para la conciliación de la vida laboral y familiar; asimismo, existe desinterés debido a la falta de reconocimiento de la especialidad.

Costa Rica cuenta con programas de maestría en enfermería en instituciones públicas y privadas. El Colegio de Enfermeras de Costa Rica dispone de un reglamento para el registro y reconocimiento de los cursos de posgrado, que comprende tanto los cursos ofrecidos en el país como los cursados en el extranjero. En el país no existe un programa de doctorado en enfermería, pero sí existen cursos en áreas afines, como educación, cultura y otras áreas que cursan las enfermeras. Otro reto es el reconocimiento gubernamental de los cursos de posgrado, que únicamente reconoce los de salud mental y psiquiatría, y el de ginecología y obstetricia; sin embargo, el título no siempre se traduce en salarios más altos y la formación implica un costo.

En México se ofrecen programas de maestría y doctorado en universidades públicas y privadas; además, algunas especialidades se han convertido en maestrías, pero aún se ofrecen cursos que otorgan el título de especialista mediante certificación profesional. Los posgrados no siempre conducen a mejores salarios, pero el curso de especialización es un requisito para acceder a algunos puestos de trabajo.

### Regulación del ámbito de práctica

En todos los países, la legislación se aplica a nivel nacional; en México existe una legislación nacional general, aunque el alcance del ejercicio profesional puede variar entre los subsistemas sanitarios y sus instituciones, así como entre los estados de la federación. El procedimiento de registro profesional y los instrumentos normativos que regulan la práctica de la enfermería se presentan en la Figura 3.

**Figura 3:** Registro profesional e instrumentos normativos de la regulación del ejercicio de enfermería en Brasil, Chile, Colombia, Costa Rica y México, 2023

**País**	**Registro profesional e instrumentos normativos**
**Brasil**	Registro profesional: Consejo Federal de Enfermería Instrumentos normativos: Ley de ejercicio profesional, n.º 7.489/1986^17^, Código de deontología profesional^18^; además de resoluciones, decisiones y dictámenes normativos del Consejo Federal y del Sistema de Consejos Regionales de Enfermería^19^, así como ordenanzas, decretos y otros documentos normativos del Ministerio de Salud y secretarías de salud.
**Chile**	Registro profesional: Colegio de Enfermeras de Chile^20^ Instrumentos normativos: Ley 19.536/1997 - reconoce el rol de la enfermera en el Código Sanitario chileno; Ley 19.937/2004 - inserta la “gestión del cuidado” en la estructura de los Servicios de Salud dependientes del Estado, entendiéndola como el rol de la enfermera^21^ ^-^ ^22^; y el Código de Ética - 2008^20^.
**Colombia**	Registro profesional: Registro Único Nacional de Enfermería en la Asociación Nacional de Enfermeras de Colombia, y, a nivel nacional, la Organización Colegial de Enfermería expide la Tarjeta Nacional de Identificación con base en la inscripción en el Registro Único Nacional del Talento Humano en Salud. Instrumentos normativos: Ley de reglamento profesional - Ley 266/1996^23^; Ley 911/2004 - Tribunal Nacional de Ética en Enfermería^24^.
**Costa Rica**	Registro profesional: Colegio de Enfermeras de Costa Rica. Instrumentos normativos: Ley Orgánica del Colegio de Enfermeras (Ley 2.343/1959) y Ley 7.085/87 - establece los diferentes niveles de enfermeras que existen en las instituciones de salud, dependiendo de la complejidad de los departamentos o servicios de enfermería y del volumen de trabajo.
**México**	Registro y licencia de profesionales se realizan junto al Gobierno mexicano. En el 2000 se instituyó la certificación de enfermería (un procedimiento voluntario que no sustituye a la licencia, ni la falta de certificación implica la pérdida de la licencia). Existen dos organizaciones con autorización oficial para la certificación: el Consejo Mexicano para la Certificación de Enfermería y el Colegio Mexicano de Licenciados en Enfermería^25^. Instrumentos normativos: Ley General de Profesiones (1945) y existen las Normas Oficiales Mexicanas (NOM) que establecen los espacios de práctica de la enfermería en el territorio nacional^25^.

Otro elemento debatido fue el desempeño de enfermeras licenciadas como auxiliares o técnicas de enfermería, una práctica presente en Costa Rica y México, mientras que en Brasil no es posible que una enfermera ejerza como auxiliar o técnica de enfermería, a menos que haya completado el curso de auxiliar o técnica y esté registrada en ese nivel.

### Iniciativas para ampliar el ámbito de práctica de enfermeras en los servicios de atención primaria de salud

El contexto de la ampliación del ámbito de práctica de las enfermeras en los países analizados es heterogéneo y depende de la fase de desarrollo de la práctica y de la formación de enfermería en cada país, así como con las estrategias de regulación de la práctica profesional, las características de los sistemas de salud y las disposiciones y normas de las instituciones de salud. A continuación, se presentan los principales aspectos por país.

#### Brasil

En Brasil, el *Conselho Federal de Enfermagem* (COFEN) y la *Associação Brasileira de Enfermagem* han creado grupos de trabajo para promover debates sobre el tema y elaborar documentos. En 2023, el Ministerio de Salud creó un grupo de trabajo integrado por diferentes asociaciones y especialidades de enfermería, que asumió un papel de liderazgo en los debates sobre la práctica en enfermería y posibilidades de ampliar el rol de las enfermeras. La investigación sobre el tema también se ha fomentado y se han publicado diversos artículos en distintas revistas científicas.

Desde 2021, el COFEN ha buscado el intercambio de experiencias internacionales para apoyar los procesos de ampliación del ámbito de práctica. La organización ha establecido alianzas con la CAPES mediante el apoyo económico a las maestrías profesionales ya existentes en Brasil y ha impulsado el desarrollo de propuestas de maestrías en EPA. 

La ley de ejercicio profesional garantiza a las enfermeras la posibilidad de ampliar su ámbito de práctica en programas de salud pública, mediante protocolos adecuados^17^. Dado que el país adopta el modelo de autorregulación de la profesión por los consejos profesionales, ello favorece la actualización de resoluciones y actos normativos que se consideran necesarios para respaldar el ejercicio profesional. 

A lo largo de los años, se ha observado una ampliación de la función de la enfermera en diversas áreas, mediante varias especialidades, como la neonatología, la obstetricia en atención secundaria y el apoyo diagnóstico por medio de ecografía. Como en la APS, la función de estas profesionales se ha ampliado a partir del desarrollo de protocolos clínicos en diversas áreas, lo que permite considerar que ya se están desarrollando prácticas avanzadas.

A pesar de este escenario, aún existen desafíos para la ampliación del ámbito ampliado de práctica: entre ellos, la formación de especialistas, la reglamentación, el mercado de trabajo y la aceptación de las EPA por parte de otros profesionales, por los servicios y proveedores de salud y de la población.

Entre los elementos que se deben analizar figuran si la EPA constituye otra categoría profesional y cómo se relacionaría con las enfermeras, que ya cuentan con un ámbito de actuación ampliado en Brasil, y la adecuación del modelo formativo, dado que existen opiniones divergentes sobre la formación a nivel de maestría o de residencia. 

#### Chile

En Chile, la implementación de la EPA se llevó a cabo mediante debates con actores internacionales e iniciativas que utilizaron el proceso participativo para la enfermería de práctica avanzada, centrado en el paciente y basado en la evidencia (PEPPA, por sus siglas en inglés)^26^, que desarrolla el programa a partir de las necesidades del país e identifica a los interlocutores para su inserción en los espacios clínicos.

La Universidad Católica de Chile asumió el desafío de crear un programa de formación en EPA y desde el principio se consideró necesario abordar los aspectos políticos para formar de manera crítica a las enfermeras. Sin embargo, los intentos de ampliar la función en la APS encontraron resistencia por parte de los médicos de familia y comunidad. Al mismo tiempo, se generaron las condiciones para ampliar la función de las enfermeras en oncología a partir del Plan Nacional del Cáncer 2018-2028, que contempla la creación de un programa de posgrado para profesionales no médicos, orientado a ampliar el ámbito de práctica^27^. Se crearon entonces dos programas de magíster de EPA en esta área.

La EPA en oncología se facilitó en el entorno clínico, ya que las enfermeras estaban implicadas y desempeñaban funciones establecidas, por lo que continuaron en los mismos puestos y funciones, aunque con una formación sólida, lo que redundó en mejoras en la atención a los pacientes. No obstante, la remuneración constituye el principal desafío, pues no se han efectuado incrementos salariales en este nuevo rol ni se han definido puestos o funciones específicas.

Existen iniciativas por parte del Ministerio de Salud para el reconocimiento de especialidades; entre ellas la EPA. Sin embargo, en el país persisten conflictos en torno a la regulación del ejercicio profesional, que requieren un debate más amplio acerca de la formación y la práctica en salud.

A pesar de estos retos, las perspectivas de ampliar el ámbito de práctica siguen siendo alentadoras y ha habido un amplio debate al respecto. Se plantea como premisa que es fundamental considerar el enfoque de la APS.

#### Colombia

Existen distintas iniciativas para desarrollar el rol de la EPA en los diferentes niveles de atención, que cuentan con el apoyo del Ministerio de Salud. Se han realizado siete encuentros sobre la enfermería de práctica avanzada, con la participación de personas que han apoyado experiencias en otros países.

Asimismo, la reforma del sistema de salud se orienta al fortalecimiento de las acciones preventivas y se ha intentado establecer el marco normativo que incluya la financiación de la formación en práctica avanzada. La prioridad es iniciar la formación en las universidades públicas y definir el perfil profesional de la EPA en la APS.

La posición del amplio grupo de formación avanzada, integrado por directores de programas de maestría y doctorado y miembros de la junta directiva de la Asociación Colombiana de Facultades y Escuelas de Enfermería, sostiene que el nivel de formación de la EPA debe ser el de maestría de profundización.

A pesar de las iniciativas, los desafíos de la implementación residen en varios aspectos, entre ellos, la oferta y el acceso a cursos, acompañados de una norma del Ministerio que permita el desarrollo de la práctica y apoye los estudios de posgrado.

Otra dificultad es el bajo reconocimiento de la formación de postgrado, que no siempre conlleva reconocimiento por parte de pares y por la sociedad, ni se traduce en niveles salariales superiores. Existen otros aspectos que favorecen la formación a nivel de maestría, como la oferta de becas que incentivan la capacitación. También se ha buscado un incentivo económico mediante el sostenimiento educativo mensual previsto en la Ley de Residentes, actualmente exclusiva para médicos. Las residencias, como parte de la formación de posgrado de enfermería, favorecerían la mejora de la práctica clínica mediante un proceso de inmersión y con salarios que permitan dedicarse a esta formación.

De forma más inmediata, se ha avanzado en la definición del perfil con el fin de orientar la implementación de la EPA. También se ha avanzado en los procesos curriculares y organizativos de los cursos.

#### Costa Rica

En el 2017, el Colegio de Enfermeras de Costa Rica celebró la primera reunión para iniciar el proceso de análisis y definición de la línea de trabajo para el desarrollo de la EPA. En 2019 se realizó el primer taller nacional de EPA, mediante un grupo de trabajo intersectorial que involucró al sector académico y buscó definir acciones a corto y mediano plazo. 

En las universidades existen iniciativas para analizar la posibilidad de ofrecer formación en práctica avanzada con grupos de enfermeras líderes en Centroamérica. En relación con la práctica de enfermería, se señala la necesidad de superar la escasez de enfermeras y la deficiente infraestructura, así como de consolidar el liderazgo de las enfermeras. 

Costa Rica también presenta un alto porcentaje de enfermeras graduadas subcontratadas por debajo de su nivel de formación. En este proceso de debate, se han identificado temas que requieren mayor profundización en el país, como la regulación del ejercicio profesional. 

Es necesario pensar en un perfil de profesional con funciones avanzadas que, de hecho, pueda responder a las necesidades de salud de la población; lo que necesariamente implica el proceso de formación y cómo se delinearán las propuestas de práctica avanzada. Por tanto, para lograr cambios estructurales, es necesario contar con un perfil profesional de enfermería no solo cualificado desde el punto de vista asistencial y de la gestión del sistema, sino también con competencias políticas que sitúen a las enfermeras en las mesas de decisión y les permitan influir en la construcción de políticas más amplias.

#### México

En el 2019 se constituyó un grupo de trabajo sobre la ampliación del ámbito de práctica de las enfermeras liderado por la OPS, la Universidad Autónoma de México, la Universidad Autónoma de Xochimilco y la Secretaría de Salud, con la participación del Colegio de Enfermeras, de la Comisión Permanente de Enfermería de la Secretaría de Salud y de las universidades.

Entre los productos se encuentra el desarrollo de un marco normativo mexicano de competencias de enfermería, con seis dominios y 68 competencias para la lista ampliada de funciones de las enfermeras con experiencia en la APS. La estrategia de México para potenciar el trabajo de enfermeras tiene dos vertientes: 1) ampliar las funciones de quienes llevan dos o más años trabajando en la APS, en la medida en que la normativa vigente lo permita; 2) establecer el rol de la EPA en el país, lo que incluye cambios normativos y formación a nivel de maestría.

La Secretaría de Salud ha asumido un papel central en todo el proceso y se prepara para lanzar experiencias piloto en varios estados de la República Mexicana, en los que se ubicará a estas enfermeras de práctica avanzada en centros de salud donde podrán desempeñar funciones ampliadas. Para ello, se han formulado propuestas de programas de formación de EPA basadas en las recomendaciones internacionales para maestrías y con un enfoque de salud pública. El primer programa de maestría se realizó entre 2021 y 2022. 

Cabe señalar que el debate en torno a la ampliación de la función de las enfermeras en México también se ha desarrollado por vías paralelas, pues ha involucrado a otros actores fuera del sistema de salud. 

Aún quedan retos por superar en este proceso, y el principal de ellos es la infravaloración de las enfermeras licenciadas. Las especialidades no son garantía de mejores salarios, por lo que resulta preocupante la exigencia de una maestría para la EPA. También es necesario avanzar en el ámbito regulatorio, incluida la consideración de las diferencias en el alcance de las prácticas ya reguladas entre los distintos estados. 

## Discusión

La formación y la práctica de la enfermería son heterogéneas en los contextos analizados. La configuración del ámbito de práctica en los países analizados se ha definido en función de sus realidades específicas. A pesar de las similitudes en el rol de las enfermeras, el alcance de sus acciones, su reconocimiento y su influencia continúan siendo dispares. 

La concepción de práctica avanzada y las motivaciones e intereses para implementarlas también varían entre países^28^. Del mismo modo, un análisis de la situación de la EPA en Europa revela importantes variaciones en la regulación, la formación y el ámbito de práctica, con diferentes niveles de competencias y autonomía en la atención de salud^29^ ^-^ ^30^.

Se ha señalado la infrautilización del potencial de las enfermeras, de su formación y capacidad para atender las necesidades de la población y contribuir a la gestión y a la formulación de políticas de salud. Esta infrautilización se manifiesta tanto en la escasez de puestos de trabajo como en la falta de reconocimiento de estas profesionales para ocupar puestos estratégicos.

Al tratarse de una función incipiente en los países objeto de estudio, se identifica una fragilidad en términos de regulación, por lo que es necesario establecer un marco normativo que reconozca formalmente la función, defina los criterios de licencia y de renovación del registro, como lo demuestra la experiencia de países en los que la función ya está consolidada^31^.

En este contexto, es fundamental sentar las bases para definir las normas de la práctica y abogar por reglamentos específicos^32^. Los procesos regulatorios deben revisarse y actualizarse con base en las necesidades de los sistemas locales de salud y en la creación de espacios laborales con protección social para los trabajadores.

Identificar y abordar las barreras regulatorias es esencial para el pleno desempeño de la práctica de la EPA y para ampliar sus contribuciones potenciales a la innovación y a la transformación de los modelos de atención^33^.

Sin embargo, los hallazgos del estudio indican que prevalece una regulación deficiente^34^, lo que puede hacer que esta titulación no sea reconocida de inmediato y, además, implica baja inserción en el mercado, falta de definición del rol, diferenciación salarial y desincentivos para seguir capacitándose, lo que se agrava, además, porque en algunos países la inversión económica necesaria para acceder a los programas de posgrado es elevada.

Es importante garantizar que las enfermeras puedan desempeñar plenamente sus funciones, de conformidad con su marco normativo, para que la labor de estas profesionales tenga un impacto significativo. Es necesario aumentar el número de puestos de trabajo, principalmente en la APS, garantizar condiciones de trabajo decentes y revisar los procesos de trabajo para reposicionar el papel de las enfermeras en el contexto de los equipos interprofesionales de salud. 

Invertir en enfermería y reconocer su función implica reducir las desigualdades de género, dado que se trata de una categoría profesional mayoritariamente femenina, cuyas integrantes se enfrentan a diferencias en las condiciones de trabajo, en las oportunidades de ascenso y liderazgo y en la remuneración^35^.

En cuanto al desarrollo de capacidades, la ampliación del ámbito de práctica requiere una formación coherente y de calidad que proporcione a los profesionales conocimientos amplios y un pensamiento crítico sobre las condiciones de salud. 

Los resultados presentados en este estudio indican distintas posibilidades de programas de formación, con énfasis en las maestrías o los programas de residencia. La experiencia internacional apunta a la adopción de programas formales de formación, las maestrías son las más frecuentes^31^.

Se recomienda ampliar los programas de formación mediante ayudas económicas públicas que permitan una mayor dedicación y reduzcan la preocupación por los ingresos familiares. 

Los países objeto de estudio presentan diferencias en cuanto a la oferta de programas de formación. La oferta y la capacidad formativa en especialización, maestría y doctorado deben optimizarse para la formación de la EPA; sin embargo, es necesario identificar y definir las competencias requeridas para estas profesionales, lo que facilitará la comprensión, la visibilidad y la implementación de la EPA en los servicios de salud^36^.

También es importante discutir la función de este profesional desde la educación y la práctica en equipos interprofesionales^37^ ^-^ ^38^. La ampliación del rol de las enfermeras, la combinación de tareas y el desarrollo de equipos interprofesionales son estrategias que pueden emplearse para hacer frente a la escasez de recursos humanos para la salud y obtener resultados positivos en la mejora de la atención a salud de la población y la satisfacción de los profesionales y los usuarios, especialmente en la APS^37^. Las evidencias indican que la integración de la EPA contribuye a mejorar los resultados asistenciales, ampliar el acceso, reducir los costos sanitarios, mejorar la calidad de la atención y aumentar el bienestar de los pacientes^39^ ^-^ ^40^.

Sin embargo, es necesario fomentar un entendimiento compartido entre los equipos de salud, reconociendo el papel y las contribuciones de la EPA, su experiencia y autonomía en la atención a la salud^41^.

La implementación efectiva y oportuna de la EPA debe basarse en las necesidades de salud y no en imperativos de la fuerza laboral ni de los profesionales. Aunque el proceso de implementación pueda ser prolongado, la experiencia de otros países en los que la función se ha implementado revela la importancia de un debate amplio y una auténtica colaboración entre los responsables políticos, los educadores, los empleadores y las asociaciones profesionales para garantizar que todas las iniciativas se ajusten a los objetivos durante su implementación^30^ ^,^ ^42^.

Asimismo, es necesario estimular la participación de estas profesionales en la formulación de políticas y en los espacios de toma de decisiones. Resulta deseable proporcionar mayor información a la sociedad sobre su labor, haciendo hincapié en la evidencia científica de sus resultados. 

Una limitación de este estudio radica en la naturaleza de los datos, basados en las percepciones y experiencias de participantes seleccionados. Sin embargo, la inclusión de diferentes actores clave en cada país y la triangulación con datos secundarios refuerzan la solidez y la credibilidad de los hallazgos. A pesar de esta limitación, el estudio aporta contribuciones relevantes al presentar un análisis comparativo de diversos contextos nacionales, lo que permite identificar factores facilitadores y desafíos comunes para la ampliación del ámbito de práctica de enfermería en la Región de las Américas.

## Conclusión

La implementación de la función de enfermera de práctica avanzada es una oportunidad real para que los países amplíen el acceso y la cobertura de salud, especialmente en la atención primaria de salud. Hay interés e iniciativas para ampliar la función de las enfermeras en América Latina; sin embargo, aún queda un camino por recorrer en términos de formación, ámbito de práctica, regulación y mercado laboral. 

El sector académico ha desempeñado un papel importante al fomentar la implementación de la EPA mediante el desarrollo de programas de formación. Sin embargo, la experiencia de los países latinoamericanos y de otros países en los que la EPA está consolidada pone de relieve el papel de los grupos de trabajo y de las acciones y programas intersectoriales, con amplia participación de diversos actores en el diálogo político para la implementación efectiva y oportuna de esta función rol.

## Data Availability

Los conjuntos de datos relacionados con este artículo estarán disponibles previa solicitud al autor correspondiente.
